# Decoding hand and wrist movement intention from chronic stroke survivors with hemiparesis using a user-friendly, wearable EMG-based neural interface

**DOI:** 10.1186/s12984-023-01301-w

**Published:** 2024-01-13

**Authors:** Eric C. Meyers, David Gabrieli, Nick Tacca, Lauren Wengerd, Michael Darrow, Bryan R. Schlink, Ian Baumgart, David A. Friedenberg

**Affiliations:** 1 Medical Device Solutions, Battelle Memorial Institute, 505 King Ave, Columbus, OH 43201 USA; 2 Health Analytics, Battelle Memorial Institute, 505 King Ave, Columbus, OH 43201 USA

## Abstract

**Objective:**

Seventy-five percent of stroke survivors, caregivers, and health care professionals (HCP) believe current therapy practices are insufficient, specifically calling out the upper extremity as an area where innovation is needed to develop highly usable prosthetics/orthotics for the stroke population. A promising method for controlling upper extremity technologies is to infer movement intention non-invasively from surface electromyography (EMG). However, existing technologies are often limited to research settings and struggle to meet user needs.

**Approach:**

To address these limitations, we have developed the NeuroLife^®^ EMG System, an investigational device which consists of a wearable forearm sleeve with 150 embedded electrodes and associated hardware and software to record and decode surface EMG. Here, we demonstrate accurate decoding of 12 functional hand, wrist, and forearm movements in chronic stroke survivors, including multiple types of grasps from participants with varying levels of impairment. We also collected usability data to assess how the system meets user needs to inform future design considerations.

**Main results:**

Our decoding algorithm trained on historical- and within-session data produced an overall accuracy of 77.1 ± 5.6% across 12 movements and rest in stroke participants. For individuals with severe hand impairment, we demonstrate the ability to decode a subset of two fundamental movements and rest at 85.4 ± 6.4% accuracy. In online scenarios, two stroke survivors achieved 91.34 ± 1.53% across three movements and rest, highlighting the potential as a control mechanism for assistive technologies. Feedback from stroke survivors who tested the system indicates that the sleeve’s design meets various user needs, including being comfortable, portable, and lightweight. The sleeve is in a form factor such that it can be used at home without an expert technician and can be worn for multiple hours without discomfort.

**Significance:**

The NeuroLife EMG System represents a platform technology to record and decode high-resolution EMG for the real-time control of assistive devices in a form factor designed to meet user needs. The NeuroLife EMG System is currently limited by U.S. federal law to investigational use.

**Supplementary Information:**

The online version contains supplementary material available at 10.1186/s12984-023-01301-w.

## Introduction

Stroke is a leading cause of long-term disability in the United States, affecting more than 800,000 people per year [1]. Unilateral paralysis (hemiparesis) affects up to 80% of stroke survivors, leaving many to struggle with activities of daily living (ADLs) including the ability to manipulate objects such as doors, utensils, and clothing due to decreased upper-extremity muscle coordination and weakness [2]. Restoration of hand and arm function to improve independence and overall quality of life is a top priority for stroke survivors and caregivers [3]. Intensive physical rehabilitation is the current gold standard for improving motor function after stroke. Unfortunately, 75% of stroke survivors, caregivers, and health care providers report that current upper extremity training practice is insufficient [4]. The development of user-centric neurotechnologies to restore motor function in stroke survivors could address these unmet clinical needs through a range of different mechanisms, such as improving motivation, enhancing neuroplasticity in damaged sensorimotor networks, and enabling at-home therapy.

Assistive technologies (AT) hold potential to restore hand function and independence to individuals with paralysis [5]. ATs, including exoskeletons and functional electrical stimulation (FES), can assist with opening the hand and also evoke grips strong enough to hold and manipulate objects [6]. Additionally, these systems have been used therapeutically during rehabilitation to strengthen damaged neural connections to restore function [7]. A wide variety of mechanisms to control ATs have been investigated including voice [8], switch [9], position sensors [10], electroencephalography (EEG) [11], electrocorticography (ECoG) [12], intracortical microelectrode arrays (MEA) [13], and electromyography (EMG) [14]. Unfortunately, no single system has simultaneously delivered an intuitive, user-friendly system with a high degree-of-freedom (DoF) control for practical use in real-world settings [4].

Recent advances in portable, high-density EMG-based (HDEMG) systems have the potential to overcome several of these barriers and deliver an intuitive and entirely non-invasive AT control solution [15, 16]. While various EMG-based ATs exist, including the commercially available MyoPro Orthosis [15], most of these systems use a small number of electrodes and rely on threshold-based triggering [14]. Consequently, these systems have limited DoF control which constrains their practical use. Conversely, HDEMG systems consisting of dozens of electrodes and leveraging machine learning approaches to infer complex movement intention can provide high DoF control, significantly expanding functional use cases as well as increasing the proportion of the stroke population that could benefit from these technologies [16–19]. Currently, HDEMG systems are primarily research systems and are not optimized for usability, including being difficult to set up, requiring manual placement of electrodes, and being non-portable and bulky, which can hinder the successful translation of technologies [4].

To address these limitations, we developed the NeuroLife^®^ EMG System to decode complex forearm motor intention in chronic stroke survivors while simultaneously addressing end user needs. The EMG system was designed to be used as a control device for various end effectors, such as FES systems and exoskeletons. Additionally, the system was specifically designed to meet user needs in domains previously identified as high-value for stroke survivors: donning/doffing simplicity, device setup and initialization, portability, robustness, comfortability, size and weight, and intuitive usage [4]. The sleeve is a wearable garment consisting of up to 150 embedded electrodes that measure muscle activity in the forearm to decode the user’s motor intention. A single zipper on one edge of the sleeve allows for a simplified and streamlined donning and doffing by the user and/or a caregiver. The sleeve design facilitates an intuitive setup process as embedded electrodes that span the entire forearm are consistently placed, eliminating the need for manual electrode placement on specific muscles. The lightweight stretchable fabric, similar to a compression sleeve, was chosen to enhance comfort for long-term use. The sleeve connects to backend Intan hardware housed in a lightweight, 8 × 10″ signal acquisition module appropriate for tabletop upper-extremity rehabilitation. Overall, these design features help address critical usability factors for ATs [4].

In this work, we demonstrate that our EMG system can extract task-specific myoelectric activity at high temporal and spatial resolution to resolve individual movements. Based on EMG data collected from seven individuals with upper extremity hemiparesis due to stroke, trained neural network machine learning models can accurately decode muscle activity in the forearm to infer movement intention, even in the absence of overt motion. We demonstrate the viability of this technique for online decoding, as two subjects used the system for closed-loop control of a virtual hand. This online demonstration is a promising step towards using HDEMG sleeves for high DoF control of ATs based on motor intention. Finally, we present usability data collected from study participants that highlight the user-centric design of the sleeve. These data will be used to inform future developments to deliver an effective EMG-based neural interface that meets end user needs.

## Methods

### Subjects

Seven individuals (3 female, 4 male; 60 ± 5 years) with a history of stroke participated in a study that recorded EMG using the NeuroLife EMG System while attempting various hand and wrist movements. Additionally, data were collected from seven able-bodied individuals (4 female, 3 male; 27 ± 1 years) to serve as a general comparison of EMG data and to benchmark decoding algorithms. Able-bodied subjects were employees of Battelle Memorial Institute, but none were authors of this work. Data were collected as part of an ongoing clinical study being conducted at Battelle Memorial Institute that was approved by the Battelle Memorial Institute Institutional Review Board. All participants provided written informed consent before participation, in accordance with the Declaration of Helsinki. Demographics of study subjects with stroke are provided in Table 1 (data on able-bodied participants can be found in Additional file 1: Table 1). Eligibility criteria were set to recruit adult chronic stroke survivors with hemiparesis affecting the arm and hand that were able to follow 3-step commands and communicate verbally. Specific inclusion and exclusion criteria are listed in the Additional file 1: Methods.

**Table 1:** Demographics of subjects with stroke and clinical metrics

Subject	UEFM	Time since stroke, years	Side of paresis	UEFM Hand	MAS Finger	MAS Wrist
13,762	36	6	Right	6	1	1
29,562	22	4	Right	2	1	1
30,458	32	3	Left	6	1	1
47,513	19	4	Right	4	1	0
61,204	8	6	Right	0	4	3
87,134	7	7	Right	0	4	4
98,473	38	6	Right	7	0	0

During the first session prior to EMG data collection, standardized clinical assessments were performed by a licensed occupational therapist in all subjects with stroke. These included the upper extremity section of the Fugl-Meyer (UE-FM) to assess upper extremity motor impairment, the Box and Blocks test to assess manual dexterity, and the Modified Ashworth test to assess spasticity of the finger, wrist, and elbow flexors. Based on predetermined exclusion criteria, an eighth subject was removed from data analysis due to hemispatial neglect affecting their ability to consistently follow movement cues.

### Experimental setup

**Fig. 1:**
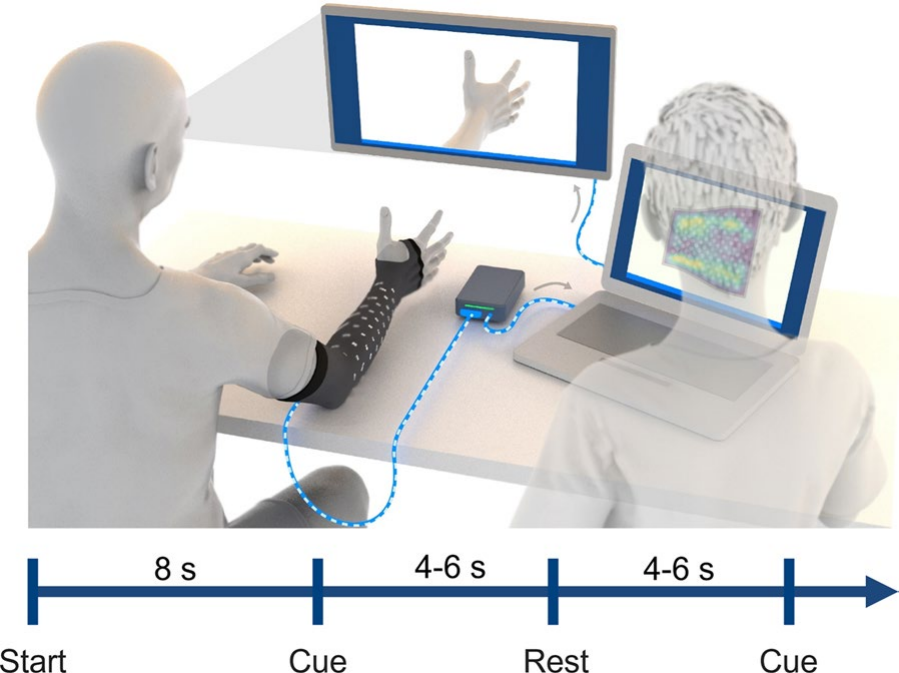
Illustration of experimental data collection procedure. Subjects were seated in front of a computer monitor with the sleeve on their impaired arm, and their arms placed on the table. The sleeve was connected to a custom-built EMG signal acquisition module, which then connected to a laptop computer. Images of hand postures were shown on the monitor and the subject followed along to the best of their ability. Each recording block was approximately 2–3 min in length and involved hand posture cues interleaved with rest periods. The recording block began with an 8-s lead in rest period. Each cue and rest period presentation time were randomly selected between 4 and 6 s for subjects with stroke. An operator ran the data collection software and observed EMG signals during data collection to ensure proper recording of data

Subjects sat facing a computer monitor with their arms placed on a table, and the sleeve on the paretic arm for participants with stroke (Fig. 1). The sleeve was placed on the right arm for able-bodied subjects, regardless of handedness. The sleeve comprises a stretchable fabric with an embedded array of electrodes (Additional file 1: Fig. S1). Depending on the forearm size of the participant, a small, medium, or large sized sleeve was used containing 128 electrodes (64 channel pairs), 142 electrodes (71 channel pairs), or 150 electrodes (75 channel pairs), respectively. Each electrode is 12 mm diameter, spaced 25 mm apart, and wrap the forearm from elbow to wrist. With a flexible and lightweight nylon-Lycra hybrid material, the sleeve wears like a compression sleeve and weighs 180, 195, and 220 g for the small, medium, and large sleeves, respectively. A zipper on the ulnar edge of the sleeve allows for easy donning and doffing. Prior to donning, an electrode solution spray (Signaspray, Parker Laboratories, Fairfield, NJ) was applied to the subject’s forearm to improve signal quality. Bipolar EMG signals were sampled at 3 kHz with a gain of 192 V/V using an Intan Electrophysiology Amplifiers (Intan RHD2000, Intan Technologies, Los Angeles, CA) [20]. An embedded electrode in the sleeve near the elbow was used as a reference for all bipolar amplifiers. The sleeve was connected to a custom-built, 8 × 10″ footprint, EMG signal acquisition module, which then connected to a laptop computer (Fig. 1 and Additional file 1: Figure S1a).

The subjects were instructed to attempt a series of hand, wrist, and forearm movements. A series of images of the desired hand movement was presented on a computer monitor, and the subjects were instructed to attempt each movement shown to the best of their ability. Subjects were instructed to attempt the movement at 25–50% of their subjective maximal effort to minimize muscle fatigue and co-contractions throughout the session.

The following movements were collected during the session: Hand Close (Power Grip), Hand Open, Index Extension, Thumb Flexion, Thumb Extension, Thumb Abduction, Forearm Supination, Forearm Pronation, Wrist Flexion, Wrist Extension, Two Point Pinch, and Key Pinch. These movements were identified by a licensed occupational therapist as highly relevant functional movements for dexterous hand use, and these movements have been used in similar studies [21]. Recording blocks consisted of a single movement repeated 10 times (referred to as “single blocks”), or multiple movements repeated within a single recording block (referred to as “mixed blocks”). Every block began with an 8 s rest period, followed by alternating movement and rest periods. During mixed blocks, a collection of movements (e.g., Hand Close, Hand Open, Forearm Supination) were randomly presented to the subject with interleaved rest periods. Before beginning the block, subjects were shown the movement(s) in the upcoming block. For subjects with stroke, the time for each movement was randomly selected from a uniform distribution between 4 and 6 s, and rest time was randomly selected between 4 and 6 s. For able-bodied subjects, the movement and rest times were both set randomly between 2 and 3 s. The cue and rest times were shortened in able-bodied subjects due to faster movement times and the expectation of simpler decoding compared to the subjects with stroke. In the last recording session, a usability questionnaire assessing user needs (adapted from [4]) was given to stroke subjects to evaluate the usability of the current sleeve design (responses from subjects are presented in Additional file 1: Table 5).

We collected data from each stroke subject across 3–4 sessions lasting < 2 h each. Data from all sessions were used to train the classifiers, with the last half of the data from the final session held out for testing. The sleeve was not doffed before collection of the test dataset in the final session. The total amount of training data per movement for subjects with stroke are shown in Additional file 1: Figure S3. For able-bodied experiments, data were collected in a single session with a total of 10 repetitions for each movement. The first 5 repetitions were used for training, and the last 5 repetitions were used for testing, without doffing the sleeve between. This structure was designed to simulate an envisioned use case in which a decoding algorithm would be calibrated for a rehabilitation session using both previous session data and data from a short same-day calibration protocol.

To assess each subject’s ability to perform the movements without any assistance, each movement was scored by a licensed occupational therapist based on a scoring scheme adapted from the Action Research Arm Test (ARAT) [18]. The “observed movement score” was ranked using the following categories: 0 = no movement; 1 = incomplete range of motion; 2 = complete range of motion but impaired; 3 = normal.

### Pre-processing, windowing, and feature extraction

**Fig. 2:**
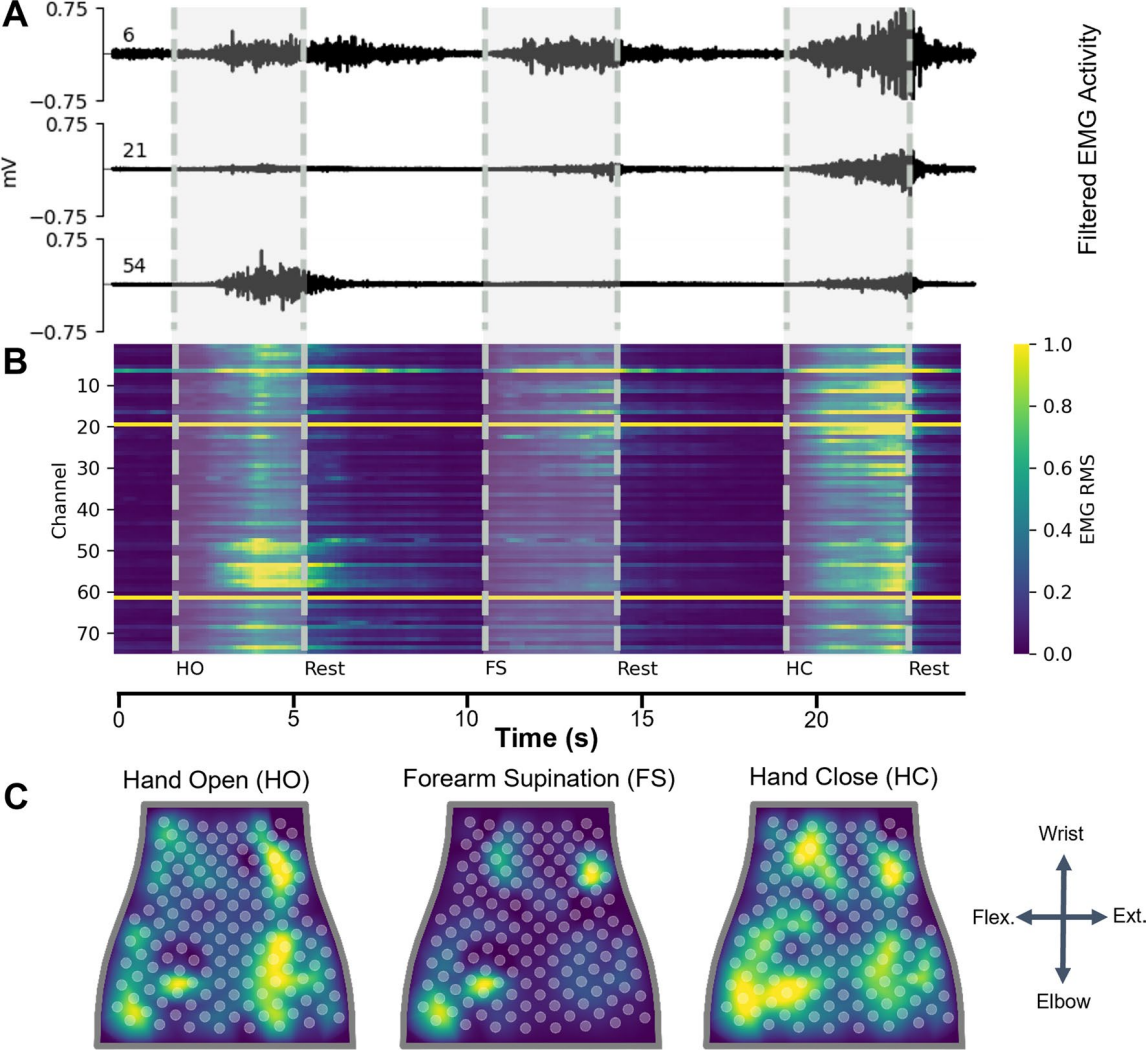
Representative EMG data recorded from subject with stroke. **A** Filtered EMG data recorded from 3 separate channels on the NeuroLife Sleeve during 3 movements: Hand Open (HO), Forearm Supination (FS), and Hand Close (HC). **B** Heatmap of normalized RMS activity, with the channel number on the y-axis and time on the x-axis. Note the activity across clusters of electrodes for each of the 3 separate movements. **C** Normalized RMS activity mapped to the sleeve orientation, with a legend showing the orientation of the sleeve mapping (flex. = flexors, ext. = extensors). Note the location of EMG activity is spatially located near the related musculature for each of the 3 movements

The EMG data were bandpass filtered (20–400 Hz, 10th order Butterworth filter), and a 60 Hz notch filter was applied similar to previous studies [23]. The root mean square (RMS) was extracted using consecutive 100 ms data windows with no overlap (Fig. 2B, C). For decoding of movement intent during a given time window, the current window and three preceding windows were used, totaling 400 ms of RMS data used for each prediction. Next, the training data were normalized (mean = 0, variance = 1) and the testing data were normalized using the mean and variance from the training data.

**Fig. 3:**
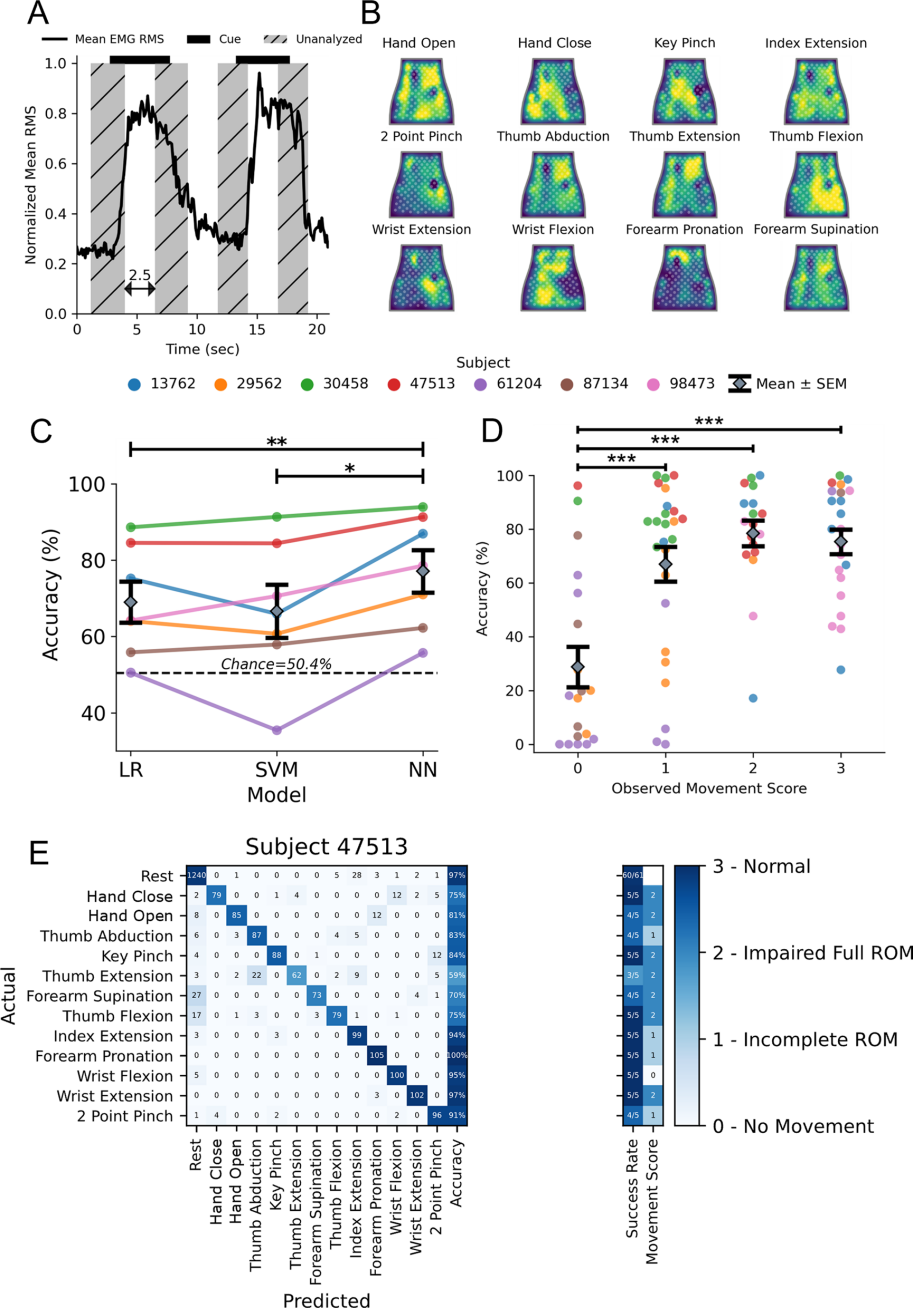
Decoding hand and wrist movements using the NeuroLife EMG System.** A** Illustration depicting the data used for training and testing the decoder. The presentation of the cue is shown as a black bar on the top of the plot, and the middle 2.5 s of the cue presentation is used for analysis. **B** Heatmaps of various movements from a subject with stroke. **C** Decoding performance comparing 3 models: LR (Logistic Regression), SVM (Support Vector Machine), and NN (Neural Network). The NN outperforms both the LR and SVM models (paired t-test NN vs. SVM, p = 9.3 × 10^–3^; NN vs. LR, p = 9.1 × 10^–4^). **D** Association between the observed movement score and decoder performance of the neural network (One-way ANOVA, Accuracy (%): F[3, 80] = 13.38, p = 3.7 × 10^–7^). The decoder struggles learning to predict movement attempts in which there was no observable movement (movement score = 0), and performs similarly when there is observable movement (movement score ≥ 1). **E** Confusion matrix for a subject with stroke detailing the decoding performance across all movements

Classification of movement intention in stroke participants was performed in two different ways: (1) using the 2.5 s center window during a cue or rest period, or (2) on the continuous timeseries data. For the 2.5 s center window method, the middle 2.5 s of each cue and rest period during a block was extracted (Fig. 3A). This resulted in a total of 22 predictions of 100 ms binned RMS data per cue (2.5 s with the first three 100 ms bins removed for containing out-of-window data at the beginning of the cue). This method was applied to both the training and testing datasets to reduce noise from motion artifact and transient muscle activation by removing the transition periods, similar to previous studies [24]. This dataset was used to evaluate different machine learning models for decoding the user’s movement intent. In able-bodied subjects, classification was performed as described above but with a 1.5 s center window during a cue or rest period, resulting in a total of 12 predictions. These data are presented in Fig. 3 for participants with stroke and Additional file 1: Figure S5 for able-bodied subjects.

For decoding of continuous timeseries data, we performed a dynamic cue shifting technique to account for the variability in the subject’s ability to respond to the onset and offset of cues. Latency between cue onset and the onset of EMG activity is a persistent problem within decoding that can lead to significant deficits in algorithm performance and is exacerbated in data recorded from subjects with neurological impairments such as stroke. Traditionally, these onset and offset variabilities are handled by shifting cues a predetermined amount of time based on reaction times [25] or by assigning each cue manually [21]. However, these methods still fail to capture the full distribution of onset and offset variability. Here, we use an automated approach to dynamically shift cue labels to match the EMG activity. The average EMG signal was aligned with the intended cue times, and residuals were calculated between the EMG signal and the signal mean for each cue segment. The transition point between segments was then iteratively optimized to minimize the sum of squared residuals (Additional file 1: Figure S4). Cue timings were shifted up to a maximum time of 2 s beyond the intended cue time.

### Classification

Classification was performed using all recording blocks (single and mixed). Importantly, the testing consisted of the final 4 recording blocks of data collected for that subject. In other words, none of the training set occurred later in time than the testing set to prevent data leakage of time dependent signal fluctuations that could significantly influence decoding performance.

Three classifiers were compared: a logistic regression (LR) model [26], a support vector machine (SVM) [27], and a neural network (NN). For the LR and SVM models, data were additionally preprocessed using principal component analysis for dimensionality reduction, keeping components that accounted for > 95% of the variance. LR and SVM models were trained using the scikit-learn toolbox [28] in Python 3.8. To optimize hyperparameters for both LR and SVM, a grid search on the training data with fivefold cross validation was applied to tailor a specific model for each subject. Hyperparameter C was varied from 1e-4 to 1e4 for LR, and hyperparameters C and Gamma were varied from 1e-4 to 1e4 for SVM. The best performing model hyperparameter combinations for each were selected for evaluation.

The NN was developed in Python 3.8 using the FastAI package [29]. FastAI defaults were used for training except where noted. The model architecture takes an input of a flattened N channels × 4 array from the N channels of the sleeve and 4, 100 ms windows of mean RMS signal. The input layer connects to two fully-connected dense layers, with size 1000 and 500 respectively, with batch normalization and the ReLU activation function between layers. The final layer had 13 classes corresponding to the 12 cued movements and rest. Finally, a Softmax activation function was applied to the model outputs to provide prediction probabilities for each of the movements. The predicted movement for a given time point was the movement with the greatest prediction probability. The training procedure used label smoothing cross entropy loss (p = 0.9) and the Adam optimizer. During training, dropout was applied to each layer with 20% probability to prevent overfitting. The learning rate was optimized using the FastAI learning rate finder tool [29]. Each model was trained for 400 epochs with early stopping criterion, using the one cycle training policy from FastAI.

**Fig. 4:**
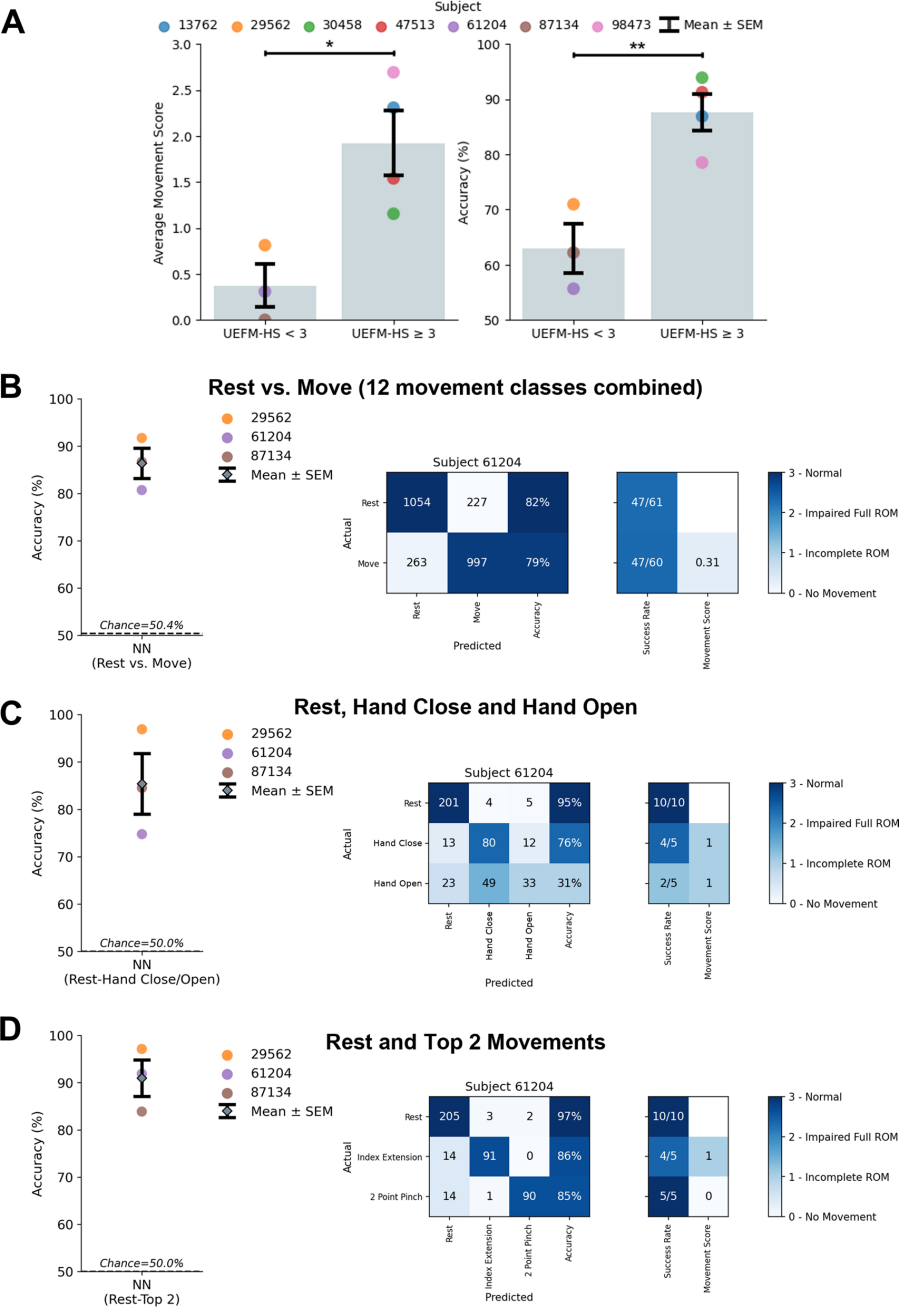
Decoding hand and wrist movements in subjects with severe hand impairment (UEFM-HS < 3). **A** *Left:* Comparison of severe (UEFM-HS < 3) and mild (UEFM-HS ≥ 3) subject impairment average movement scores (Average movement score: unpaired t-test UEFM-HS < 3 vs. UEFM-HS ≥ 3, p = 0.02). *Right:* Comparison of NN decoding performance for severe and mild subject impairments (Decoding accuracy: unpaired t-test UEFM-HS < 3 vs. UEFM-HS ≥ 3, p = 0.006). **B** Decoding performance of NN binary classifier for UEFM-HS < 3 subjects comparing Rest and Move in which Move is made up of combining all 12 movements into a single class. Confusion matrix of subject 61,204 for the two-class problem. The observed movement score is the average of all movements observed movement scores. The two-class decoder can reliably distinguish the difference between a resting and moving state. **C** Decoding performance of NN model when restricting classes to Rest, Hand Close, and Hand Open. Confusion matrix of lowest performing subject (61,204) for the three-class problem. The three-class decoder is not sufficient to distinguish the movements reliably. **D** Decoding performance of NN model when restricting classes to Rest and the top 2 movements for each subject for a total of three classes. Confusion matrix of subject 61,204 for the three-class problem. Focusing on movements specific to subjects increases the robustness of decoder performance

To simulate massed practice rehabilitation exercises, participants repeated movements with interleaved rest. We evaluated the decoding algorithms with two complementary metrics relevant to this use case and commonly used for similar applications. Accuracy was defined as the percentage of 100 ms time bins predicted by the classifier to be the same as ground truth similar to our group’s previous decoding study [30]. Accuracy is a standard classification metric and provides a high temporal resolution metric of performance. Chance level accuracy was determined based on the percentage of labels equal to the majority class (Rest; 50.41%), which represents the accuracy of a naïve classifier. Since the majority class prediction yields the highest chance level accuracy of any random strategy in a 13-class problem (e.g. stratified, uniform, or majority), we chose to use this method to represent the naïve decoder option for all reported chance levels. When decoding a subset of the full 12 movement set (Fig. 4), the rest cues directly before each target movement were sampled to maintain rest at 50% of the sampled dataset to avoid biasing the results. We also present success rate as a decoding performance metric, similar to previous studies [25]. A movement is considered successful if there is at least 1 s continuous period within a cue that is correctly decoded as the intended movement. The success rate is then calculated as the percentage of cues which are considered successful. This metric approximates an observer rating each cue as a binary success or failure and is more aligned with how a user would perceive performance.

### Real-time demonstration

In separate sessions, we tested the performance of the decoder online in two stroke subjects (Subjects 13,762 and 30,458) to demonstrate the ability to predict a user’s motor intention in real-time. An occupational therapist identified a bottle pouring task as an appropriate massed practice therapy task shared for both of these subjects based on their personal abilities. The movements Hand Open, Hand Close, and Forearm Supination were further chosen by the therapist as movements for which the NeuroLife EMG system may be programmed to control FES or an exoskeleton to assist the subjects. We used a decoder trained to classify these movements and rest for this real-time evaluation of a simulated use case. The same NN architecture described above was used during this real-time demonstration. Data collected from this online decoding session is referred to as the real-time demonstration dataset. The NN decoder was built using two blocks of training data that was collected during the online decoding session. Each block contained 5 repeats of three movements (Hand Close, Hand Open, Forearm Supination) with interleaved rests. EMG data was filtered using identical filters as the offline method described above. RMS was extracted using consecutive 100 ms data windows with no overlap, and the current window and three preceding windows were used, totaling 400 ms of RMS data for each prediction. Training data were normalized (mean = 0, variance = 1) and the online data were normalized using the mean and variance from the training data. Cue labels were shifted by 300 ms to account for reaction time of the participant. To avoid unintentional flipping between states in the online system, the NN class output probabilities needed to exceed a threshold of 0.6 to change the decoder class prediction from the previous prediction. Additionally, a stable decoder output was required to change decoder state, therefore two consecutive samples of the same prediction class were required to update the final class prediction. An experimenter then prompted each subject with randomized cues with the online decoder running. A virtual hand on the computer screen reflected the real-time movement detection. An experimenter manually labeled cues provided to the participant and the decoding accuracy was calculated. Videos of these blocks for each subject can be found in Additional file 1: Media 1 and 2. Following the online session, NN models were re-trained on the same real-time demonstration dataset using the same methods described above for a comparison of online and offline performance. These data are presented in Fig. 6.

### Statistical analysis

**Fig. 5:**
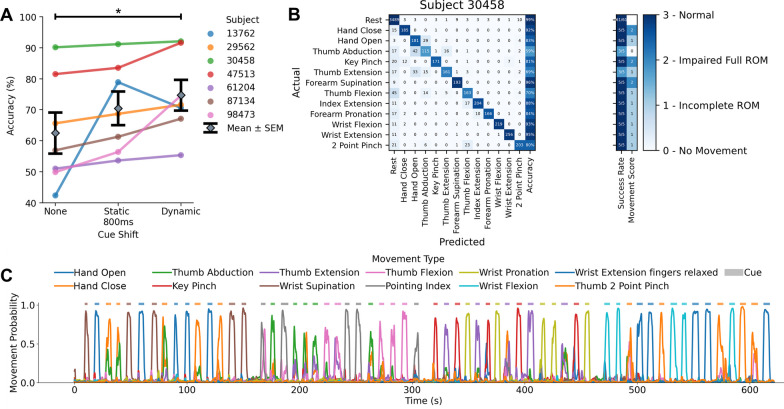
Decoding hand and wrist movements in a continuous EMG dataset. **A** Dynamic cue shifting significantly improved accuracy compared to no cue shift (Cue shift: paired t-test Dynamic vs. None, p = 0.020). There was no significant difference between a dynamic cue shift and static 800-ms cue shift (approximately the average cue shift across subjects) (Cue shift: paired t-test Dynamic vs. Static 800 ms, p = 0.22). **B** Confusion matrix detailing performance from one subject in the continuous dataset. **C** Time series plot depicting decoder class probabilities across time. The presented cue is shown in above the time series plot as a rectangular colored bar with the color corresponding to the movement class

All comparisons were planned in the experimental design a priori*.* Normality of distributions were tested using Lilliefors tests. Significant differences were determined using paired t-tests (Fig. 3C) and unpaired t-tests (Figs. 3D, 4A) and where appropriate. Significant differences for multiple comparisons were determined using one-way ANOVAs followed by Tukey HSD tests (Fig. 3C, D). Alpha of 0.05 was used for single comparisons. To correct for multiple comparisons, a Bonferroni-corrected alpha of 0.0167 was used for Fig. 3D and an alpha of 0.025 was used for Fig. 5A. The p-value for the correlations were determined using Wald Test with t-distribution of the test statistic (Additional file 1: Figure S10). Statistical tests for each comparison are noted in the text. Statistical analysis was performed in Python 3.8 using SciPy and Statsmodels. In all figures, * indicates p < 0.05, ** indicates p < 0.01, and *** indicates p < 0.001. Error bars indicate mean ± SEM in all figures.

### Usability assessment

Usability is a critical factor in the long-term adoption of an AT. Inconveniences of setup and comfort, as well as frustrations with reliability can often lead to eventual device abandonment. Therefore, in our final EMG recording session with each participant, we collected initial usability data of the NeuroLife Sleeve for use in chronic stroke survivors to help guide future development efforts. The questions posed to subjects here were adapted to investigate overarching themes mentioned by stroke survivors, caregivers, and HCPs for the use of an assistive technology [4]. Subjects answered each question on a 1 to 5 scale, and questions were targeted at the following categories: simple to apply, comfort for long-term use, freedom of movement during use, functionality / lightweightness and portability, potential for clinical and home use, and overall aesthetic design of the device (Additional file 1: Table 5). Subjects were instructed to consider a use case in which the NeuroLife EMG System (sleeve and signal acquisition module) is used to control a FES or exoskeleton system when responding. For usability metrics with more than one question (e.g. simple to apply), the mean value was scored for that assessment.

## Results

### Movement intention can be inferred from forearm EMG activity of subjects with stroke using the NeuroLife EMG system

Removing the transition periods and focusing on periods of consistent activity yielded a standardized dataset to compare performance of various models (Fig. 3A). Heatmaps of EMG activity across the sleeve are shown for one subject with stroke (Fig. 3B). These heatmaps highlight the visual differences between forearm EMG activity across the various movements. In contrast to the heatmaps of able-bodied subjects (Additional file 1: Figure S2), EMG activity is less localized in the heatmaps of subjects with stroke. This trend is consistent across stroke severity, with more severely impaired subjects having less localized forearm EMG activity (Additional file 1: Figure S9). These results are consistent with previous reports of lack of independent muscle control following stroke [31].

To validate our decoding pipeline, we tested decoding performance in able-bodied subjects across all 12 movements with the expectation of highly accurate decoding using three different approaches. Overall, the NN obtained 96.8 ± 0.5% accuracy and outperformed LR and SVM models, which had 91.5 ± 0.8% and 90.8 ± 1.2% accuracy, respectively (Additional file 1: Figure S5; One-Way ANOVA: F[3, 10] = 4015, p = 9.02 × 10^–16^; paired t-test NN vs. LR, p = 5.8 × 10^–5^; NN vs. SVM, p = 1.6 × 10^–3^). These decoding results are consistent in the dataset comprised of subjects with stroke attempting all 12 movements, where the NN obtained 77.1 ± 5.6% accuracy, and outperforms the LR (69.0 ± 5.4%) and SVM models (66.6 ± 6.9%) (Fig. 3C; One-Way ANOVA: F[4, 12] = 64.02, p = 5.41 × 10^–8^; paired t-test NN vs. LR, p = 9.1 × 10^–4^; NN vs. SVM, p = 9.3 × 10^–3^). In summary, the NN outperforms the LR and SVM when decoding forearm EMG activity to infer movement intention. All subsequent analyses were performed in subjects with stroke using the NN for decoding.

Next, we investigated the relationship between the subject’s ability to perform a movement unassisted and our ability to accurately decode that movement. Generally, decoding performance improved as the observed movement score increased (Fig. 3D; One-way ANOVA: F[3, 80] = 13.38, p = 3.7 × 10^–7^). A comparison of decoding accuracy based on movement score was computed using a Tukey HSD test (Additional file 1: Table 3). For movements with visible motion (score ≥ 1), the overall decoding accuracy was 85.7 ± 3.2%, whereas for movements where the subject had no visible motion (score = 0) the accuracy dropped significantly to 27.3 ± 3.2% (Chance: 4.0%) (Movement Ability: Movement score = 0 vs. Movement score = 1–3: unpaired t-test, p = 3.9 × 10^–9^). We also investigated the relationship between decoding accuracy and the assessed clinical metrics (Additional file 1: Figure S10). We observed moderate and significant correlations (Wald Test) between decoding performance and the UEFM Hand subset, and both the MAS wrist and fingers scores. In summary, these data suggest that EMG decoding performance decreases as impairment increases across a variety of clinical metrics assessing various aspects of dysfunction.

Next, we investigated decoding performance of individual movements in subjects with stroke. The confusion matrix with individual movements for one subject is shown in Fig. 3E. The best performing movements across subjects were Wrist Flexion and Index Extension with an average accuracy of 68.7 ± 2.2%. On average across subjects, the worst performing movements were Forearm Supination and Thumb Abduction, with an average accuracy of 39.4 ± 9.9% (Additional file 1: Figure S6). The success rate per movement type for one subject is presented in the right column of the confusion matrix (Fig. 3E). The overall grand average success rate across all movements and rest achieved 75.9 ± 4.2%. The top movements quantified by successes/attempts were Index Extension: 23/30 Wrist Flexion: 20/30, and the bottom movements were Forearm Supination: 14/43 and Thumb Abduction: 17/43.

### Decoding movement subsets to achieve high performance in subjects with severe stroke impairments

As the decoding performance of our algorithms was dependent on the presence of visible movement in our subjects, we next investigated the association of hand impairment severity based on the Upper Extremity Fugl-Meyer Hand Subscore (UEFM-HS) with observed movement scores and decoding performance (Fig. 4A). Both the observed movement score and decoding performance in subjects with severe hand impairment (UEFM-HS < 3) were significantly different than in individuals with moderate or mild hand impairment (UEFM-HS ≥ 3) (Average movement score: unpaired t-test UEFM-HS < 3 vs. UEFM-HS ≥ 3, p = 0.02; Decoding accuracy: unpaired t-test UEFM-HS < 3 vs. UEFM-HS ≥ 3, p = 0.006). To better understand if a smaller subset of movements could be decoded in the presence of severe impairment, we assessed if sufficient signal was present to decode general muscle activity during cued movement periods compared to rest. Practically, this decoding scheme would enable an individual with severe hand impairment to control an AT with a single movement. We separated the problem into two classes (Rest vs. Move), where the “Move” class consists of the 12 different movements combined into one class (Fig. 4B). The NN decoder was able to achieve high performance in individuals with severe hand impairment with 86.7 ± 2.6% accuracy and 85.2 ± 3.6% success rate (Successes/Attempts; Rest: 164/185, Move: 151/185). These results indicate that the surface EMG collected from individuals with severe hand impairment is sufficient for binary scenarios.

Encouraged by the binary decoder performance, we extended our analysis to include key functional movements for restoring grasp function, namely Rest, Hand Close, and Hand Open (Fig. 4C). In this 3-class scenario, the rest periods before each movement were downselected from the full 12 movement dataset to keep chance accuracy decoding at 50%. With these key movements in individuals with severe hand impairment, the decoding performance achieved 85.4 ± 6.4% accuracy and 88.0 ± 7.7% success rate (Successes/Attempts; Rest: 45/46, Hand Close: 22/23, Hand Open: 14/23). While decoding the movements to enable Hand Close and Hand Open is ideal for intuitive control of an AT, alternatively decoded movements with the greatest performance can be mapped to the most impactful functional movements. Thus, we tested decoding only the top performing movements for each subject (Fig. 4D). When comparing Rest and the top two movements for each individual, decoding performance achieved 91.0 ± 3.9% with a grand average success rate of 90.6 ± 4.2% (see Additional file 1: Table 4 for full details). This performance was comparable to the decoding performance of individuals with UEFM-HS ≥ 3 on 12 movements (87.6 ± 3.4%) and provides a reasonable alternative for subjects with more severe impairments.

### Decoding continuous forearm EMG data in real-time scenarios in chronic stroke survivors

To demonstrate the utility of the NeuroLife EMG System to interpret muscle activity from the forearm to act as a control signal for assistive devices, we tested our decoding algorithms in a continuous dataset. Following a stroke, the ability to contract and relax muscle groups is slowed and highly variable [32], which consequently makes automated labeling of cues using a static time shift (e.g., 800 ms) for training machine learning models imprecise. To account for this cue onset and offset variability, we first performed a dynamic cue shifting technique to automatically shift cue labels to match EMG activity (Additional file 1: Figure S4A). An average of 843 ± 95 ms of cue data per cue change or a grand average of 16.1 ± 1.0% of the full cue data stream across all subjects was shifted using this technique (Additional file 1: Figure S4B). To verify this method, we compared decoding performance with and without cue shifting. Dynamic cue shifting significantly improved decoding performance achieving 74.7 ± 5.0% overall with no cue shift achieving 62.5 ± 6.7% (Fig. 5A; Cue Shift: paired t-test Dynamic vs. None, p = 0.020). However, we found no significant difference in decoding accuracy between dynamic cue shifting and a static 800 ms cue shift (70.5 ± 5.4% decoding accuracy) representing an estimate of the average dynamic shift (Fig. 5A; Cue Shift: paired t-test Dynamic vs. Static 800 ms, p = 0.22). One subject (13,762) had an increase in decoding performance from a static shift, while the rest of the subjects experienced a decrease or no change in performance, suggesting that the dynamic cue shift was the most robust technique for our analyses.

Using the dynamic cue shifting technique, we investigated decoding performance of individual movements in the continuous dataset. The confusion matrix with individual movements for a single subject is shown in Fig. 5B. The best performing movements across subjects were Wrist Flexion and Wrist Extension, with an average accuracy of 61.2 ± 5.0%. The worst performing movements across subjects were Forearm Supination and Thumb Abduction, with an average accuracy of 29.5 ± 9.0%. A continuous time series plot of all movement probabilities is shown in Fig. 5C Shaded regions indicate the cued movement with the probability of the movement type decoded based on motor intention.

To assess whether the NN decoder could be used in real-time situations, inference testing was conducted using a Surface Book 2 with NVIDIA GeForce GTX 1060 GPU. The NN decoder was trained using cued movement data collected at the beginning of the session, totaling 1526 sample bins (2.54 min) for subject 13,762 and 3000 sample bins (5.0 min) for subject 30,458. The trained NN decoder was exported and loaded in using the Open Neural Network Exchange (ONNX) Runtime [33] for inference testing. NN forward model prediction times on average took less than 1 ms (307 ± 49 µs). Taking the entire preprocessing pipeline into consideration in addition to the NN forward prediction, the total inference time was 23.1 ± 4.4 ms. Since the resulting inference time is under 100 ms (time bin for RMS feature calculation), the NN model was deemed suitable for real-time inference.

**Fig. 6:**
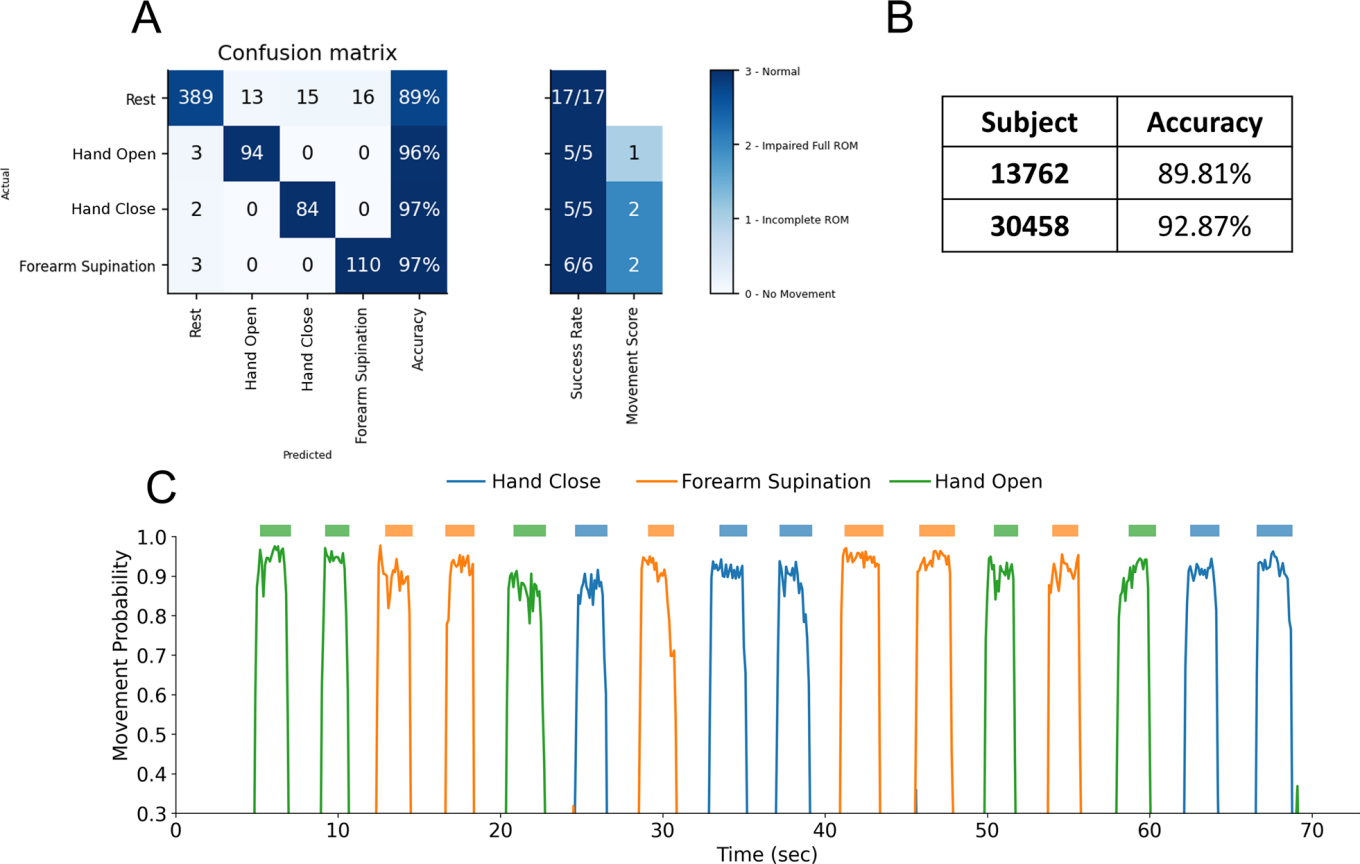
Online decoding of hand and wrist movements. **A** Confusion matrix of subject 30,458 from the online decoding session. **B** Online decoding performance for both subjects on the real-time demonstration dataset. **C** Time series plot depicting decoder class probabilities across time for subject 30,458. The presented cue is shown above the time series plot as a rectangular colored bar with the color corresponding to the movement class

We next tested the decoder online to verify closed-loop control of a virtual hand on two stroke subjects (13,762 and 30,458) using the NN model. The confusion matrix with individual movements tested during the online testing for Subject 30,458 are shown in Fig. 6A. The best performing movement was Forearm Supination, with an overall accuracy of 97.1%. The continuous time series plot of movement probabilities for Subject 30,458’s online decoding session is shown in Fig. 6C. Finally, videos from the online sessions are shown in Additional file 1: Media 1 & 2, with the user following along with movements cued from an experimenter, and the decoded motor intention controlling a virtual hand on the computer monitor. These videos demonstrate the online decoding accuracy and responsiveness of the system and highlight the utility of the NeuroLife EMG System for eventual closed-loop control of upper-extremity devices.

### The NeuroLife Sleeve meets usability needs of chronic stroke survivors

**Fig. 7:**
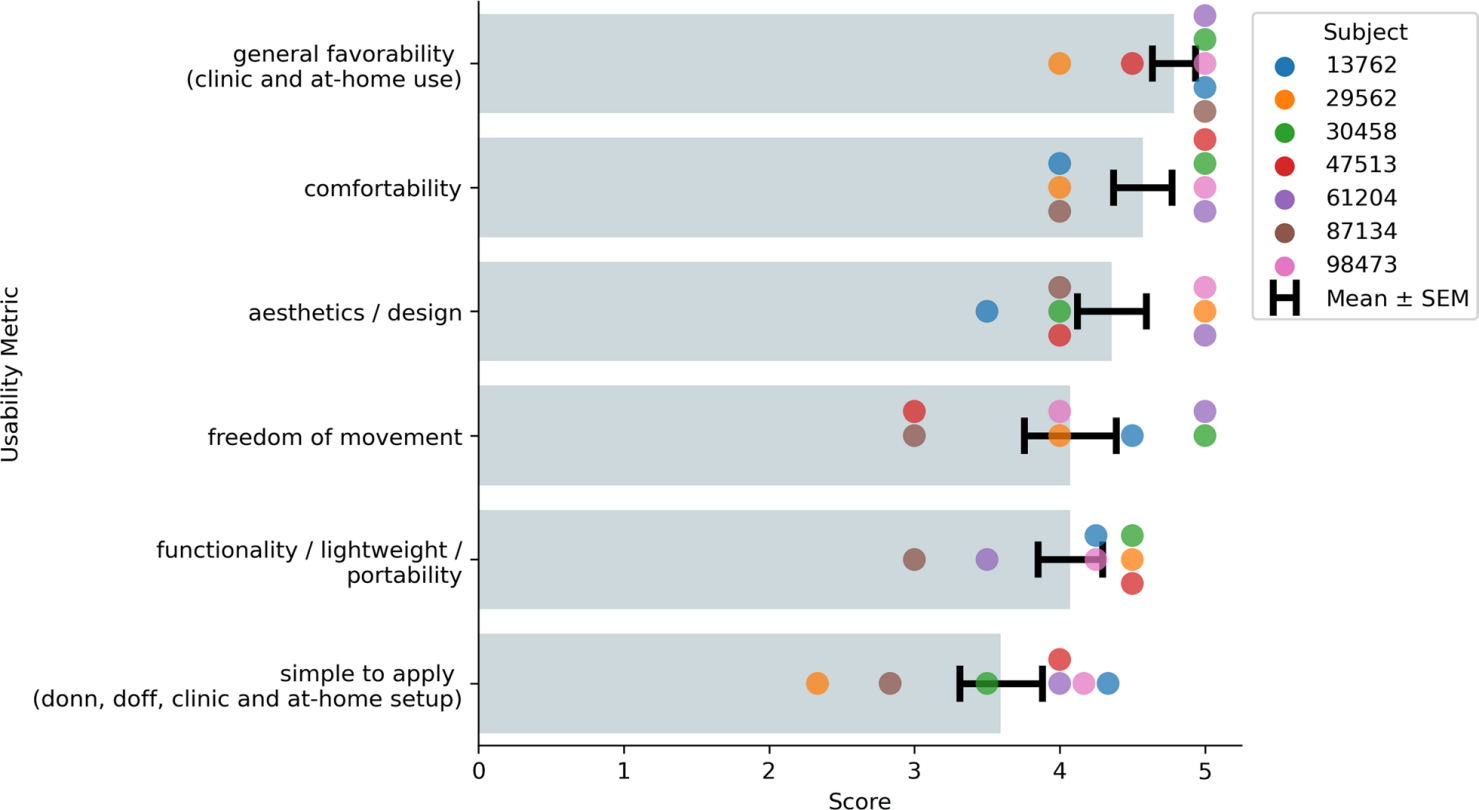
Summary of the NeuroLife Sleeve usability data from subjects with stroke. Each subject with stroke ranked the NeuroLife Sleeve based on 6 usability domains. Group data is presented for each of the 6 domains

Summary data from the usability questionnaire suggest that the NeuroLife Sleeve meets many user needs (Fig. 7). Subjects answered questions on a scale of 1 to 5 with higher values indicating stronger agreement. In general, subjects were optimistic that they could don and doff the NeuroLife Sleeve with the help of a caretaker in their home (3.60 ± 0.28). Concerns were generally centered around the pre-application of the conductive spray and relative positioning of the system, which we are actively addressing in our next design iteration. During sessions, subjects had the sleeve donned for > 1.5 h, and all participants reported general satisfaction with the overall comfort of the device (4.57 ± 0.20). The sleeve was designed with a lightweight stretchable fabric, and participants were generally satisfied with the ability to move their arm while the sleeve was donned (4.07 ± 0.32). Subjects were highly confident (4.07 ± 0.22) that they could wear the sleeve during functional light activities around their home, suggesting that the sleeve is non-restrictive, lightweight, portable, and promising for home use. A commonly overlooked barrier to widespread adoption of assistive technologies is user acceptance of the overall look and feel of the device [4]. All subjects were extremely satisfied with the overall design of the sleeve (4.36 ± 0.24). In general, they were all very excited for the opportunity to use the sleeve with the “general favorability” metric receiving the highest score of 4.79 ± 0.15. In summary, the usability results from the current study provide promising early data that the NeuroLife Sleeve can meet end user needs with directions on where to improve for future iterations.

## Discussion

In this study, we demonstrate decoding of motor intention using the NeuroLife EMG System in people with upper-extremity hemiparesis due to chronic stroke. Based on high-density surface EMG data collected from the forearm, 12 functional hand, wrist and forearm movements were classified with high accuracy. Overall decoding accuracy was associated with the subject’s ability to perform the movement (quantified here as observed movement score), with greater functional movement corresponding with higher decoding accuracy. Even in movements with little to no movement capacity (movement score ≤ 1), the system was able to accurately differentiate movement intent, albeit with some decrease in performance. Furthermore, we report that decoding performance was associated with a variety of different aspects of impairment such as overall motor impairment and spasticity. We also demonstrate online decoding of 3 task-relevant movements and rest for closed-loop control of a virtual hand, highlighting the decoding accuracy, speed and responsiveness of the system. Usability data demonstrated that the sleeve is comfortable and lightweight, allowing stroke survivors to wear the sleeve for extended periods of time without restricting their movement. In summary, this work demonstrates the NeuroLife EMG System’s utility as a wearable, user-friendly device to infer movement intention in stroke survivors with severe motor impairments.

Previous studies have demonstrated decoding of motor intention using surface EMG in the upper extremity in chronic stroke survivors [21, 34–36]. In these studies, a range of machine learning techniques, impairment levels of the participants with stroke, and types of movements were investigated. The classification accuracy we measured was comparable to previous work with similar movement sets, although differences in study methodology restrict direct comparison. We found that a NN model outperformed the LR and SVM models in both able-bodied and stroke subjects across all movements. However, decoding accuracy decreased in stroke subjects with severe motor impairments. Specifically, we find our hardware and NN decoding techniques provide high performance in able-body (96.8% accuracy) and stroke (85.7% accuracy) participants if visible movement was observed. Included in the 85.7% accuracy are movements where participants had incomplete or impaired range of motion, indicating that we could consistently decode the subject’s intent to move despite their inability to properly complete the movement. These movements would be strong candidates for improvement with an EMG-controlled assistive device. Our complete 12-movement survey is helpful for understanding what movements may be decodable for each subject and may be appropriate for facilitating ATs in individuals with moderate or mild hand impairments. However, those with severe hand impairments are unlikely to be able to accurately control that many movements. Instead, it may be desirable to use only a subset of movements customized to the individual, that they can accurately control.

A dynamic cue shifting technique may present a more robust and automated solution to account for differences among subjects. Improvement in decoding performance from using dynamic cue shifting is likely due to: (1) improved accuracy of the timing of cue onset and offsets in the training data which gives a better representation of each movement and thus better decoding performance, and (2) more accurate testing alignment and better testing parameters. These results suggest that cue labeling can substantially affect overall decoding performance in online decoders, and intelligent cue labeling can improve overall performance. Though we only briefly assessed our system’s online decoding capabilities, our initial results suggest that online EMG decoding of motor intention is possible, though more subjects and functional movements are needed to increase robustness.

Recent studies have shown encouraging results using a limited set of manually placed electrodes, which may account for some performance differences compared to our results [21, 35]. Moreover, localizing electrodes to muscle activity critical to grasp production can be an effective strategy to minimize system complexity. The optimization of electrode placement and reduction of hardware complexity is a planned future direction for the NeuroLife Sleeve. Additional studies have used similar numbers of channels as we have presented [21, 37, 38]. However, the system presented here streamlines system setup with a single zipper closure aligned by easily recognizable anatomical landmarks, the elbow and radial styloid process. Usability around donning/doffing is a key concern for adoption of AT, and systems with extensive setup procedures risk poor acceptance in clinics, rehabilitation settings, and the home. Prior studies have also shown that time domain features, such as RMS, combined with NN approaches can outperform more classical statistical or machine learning approaches [21]. Our results agree with these findings, further supporting that high density EMG recordings have sufficient complexity to leverage the recent developments in deep learning. We extend the findings of previous studies by presenting an easy-to-don and doff wearable device that removes the need for manual placement of electrodes. This reduces the necessary setup time and ensures consistent placement of recording electrodes across sessions. Additionally, we present data to support the real time performance of the decoding paradigm. This study provides evidence that the device can decode motor intention with high performance across a variety of subjects, and we demonstrate decoding speed that is fast enough to reliably perform real-time inference alongside data collection. Notably, processing in this context did not involve removing transition periods during training or online testing, indicating the system’s robustness to motion artifact. Finally, we present a viable, automated cue shifting method that removes the necessity for manual relabeling and improves system performance.

Usability is an important factor for clinical technologies to assist with stroke rehabilitation by supporting motivation for consistent and active training. While existing AT solutions show promising results, these systems tend to focus on the technology and often fall short in the user-centric designs. Most clinical ATs involve manual placement of patch electrodes and long calibration procedures which limits the amount of practice that can be achieved within a given rehabilitation session. Furthermore, many systems are bulky and lack portability, which can limit patient adoption for use outside of rehabilitation training and into the home [39]. The system evaluated here uses a lightweight wearable and reusable sleeve connected with a ruggedized cable to an 8 × 10″ signal acquisition module. Further work is required to ensure this system’s reliability a variety of home and non-laboratory contexts, but here we demonstrate that the NeuroLife EMG System can address many usability concerns of current technologies while providing robust decoding of motor intention. In combination with soft exoskeletons or FES, the sleeve can drive intention-based training coupled with functional movements in a user-centric form factor.

Based on user feedback from the current study, the sleeve design meets various end user needs. The design allows for use on either arm, and the stretchable, lightweight fabric design was reported by participants to be comfortable without limiting natural arm movements. Aesthetically, subjects were pleased with the sleeve design and advocated that they would use the system at home for rehabilitation and activities of daily living given the opportunity. Participants mostly agreed that the sleeve was straightforward to don and doff during the study with the help of the researchers and believed that they could apply the sleeve with the help of a caretaker. However, participants identified the simplicity to apply the sleeve as an area that is currently lacking, and participants were not confident in being able to apply the sleeve independently without assistance. This is an identified area for future development and will be the focus of next design iterations to enable at-home use. Despite this current usability limitation, participants indicated that not only would they feel comfortable performing rehabilitation therapy at home but are excited for the possibility of using the sleeve as a therapy tool indicated by the highest score for general favorability.

This study expands the scope of previous EMG decoding studies by presenting the performance of a novel algorithm across a wider range of subjects, UEFM score of 7 to able-bodied, in offline and online contexts while highlighting the importance of usability. The data collection was designed to simulate a realistic use case in which EMG-controlled ATs are used to assist in tabletop task-oriented upper-extremity rehabilitation. This study indicates the practicality and usability of AT control using this EMG system and highlights the shortcomings of decoding in severely impaired subjects and low observed movement scores. These findings will inform future work for the field of EMG decoding and may inspire new approaches for EMG-controlled ATs in the space of rehabilitation suitable for severely impaired stroke survivors.

The present study provides an initial demonstration of the NeuroLife EMG System to decode motor intention in chronic stroke survivors while simultaneously meeting needs, but some limitations merit consideration. We did not age match the able-bodied subjects to the stroke subjects, which may have affected comparisons between the two groups of subjects. Data was not collected from the non-paretic arm in the stroke subjects, although we do provide data from able-bodied subjects to demonstrate high-accuracy decoding to validate our approach. While the reported results indicate that the Neurolife EMG System can be used to decode motor intention in a package that meets end user needs, there is still room for improvement in various areas, including refinement of decoding algorithms, the sleeve design and related hardware, and eventual applications. Future work refining decoding algorithms will focus on overall improvements to decoding performance by leveraging many of the advancements made in recent years in the field of deep learning [40]. We will investigate the use of more sophisticated neural network models, including recurrent neural networks (RNNs), transformers optimized for time series modeling which could improve overall decoding accuracy, specifically for participants with limited movement capability [41, 42]. Advanced neural network models may also aid in our ability to identify altered states of muscle activity, including spasticity and fatigue [43, 44]. To better address inter-session variability, we will apply various machine learning techniques including unsupervised learning, data augmentation, and domain adaptation [45–48] to fully leverage multiple datasets to reduce setup and calibration times for new users. This study used about 30 min of intrasession data for training of 13-class decoders, but with further development of these techniques, similar performance may be achieved with a much shorter decoder recalibration sequence after an initial training session. We demonstrated high performance using only 3 min of training data in a 4-class online decoding scenario, which may be acceptable for some use cases. Our current model architecture does not consider the spatial information available in the sleeve. A future direction for feature extraction and decoder architecture is to include features that capture this relational data between electrodes to create decoders that are less sensitive to positional changes, such as convolutional, transformer, or graph neural networks. Improvements to data quality itself can be accomplished with visual reinforcement to subjects. An online decoding system that displays the decoded intention may be more beneficial to subject engagement over the image cues used in the current study. While we provide the initial proof-of-concept demonstration of the NeuroLife EMG System here, the data collected during the study was not representative of how the system will be ultimately deployed as an assistive device. For example, in the current study, subjects kept their elbow stationary on the table during movements and did not interact with objects, both of which can significantly influence forearm EMG activity and thus decoding performance. Future studies will focus on capturing training data in more complex situations, such as during reach and grasp tasks and object manipulations, to develop decoders robust to movement such as the spatial decoders described above. We also assumed a class distribution based on the target use-case of occupational therapy in which Rest periods occur in between movements (~ 50% of the time). However, this method oversamples the Rest class, which can mask poor performance of other movements which are more functionally relevant thus limiting comparisons to other decoding studies. For this study, we determined the chance level using a naïve decoder (i.e., always predicting the majority class). We acknowledge that this baseline is dependent on the class distribution, thus we also present the success rate metric which is designed to approximate a therapist judging binary success for each movement cue (and ignoring rest periods). Future work will examine different decoders and metrics, including those decoders presented here in Fig. 3C. Similarly, the decoding performance presented here was in the absence of assistive device control. Commonly used assistive devices, including FES and exoskeletons, may interfere with EMG activity when active and thus can significantly affect decoding performance [34, 49]. Our group is working to integrate FES functionality within the same EMG recording electrodes to eliminate the need for additional hardware, such as an exoskeleton or additional patch electrodes. Future work from our group will focus on developing algorithms that can decode EMG during FES activity. Furthermore, integration with assistive technologies will change the sleeve form factor as well as the backend hardware. The usability assessment in this study focused primarily on the sleeve component of the EMG system. Future work will include optimization of the backend hardware for space efficiency and portability with studies evaluation of the complete system usability. With a technology that incorporates EMG and FES into a single consolidated sleeve, the system has the potential to help support motor recovery and assist in ADLs [14, 25].

## Conclusion

The focus of this study was to validate the NeuroLife EMG System by decoding hand, wrist, and forearm movements and collect usability data from subjects with stroke. We demonstrate accurate EMG decoding of 12 different movement classes with a neural network in both able-bodied and stroke subjects. Decoding accuracy in stroke subjects was associated with the movement ability of each subject. The decoding results were consistent with similar myoelectric intention-based studies. We demonstrate online decoding and closed-loop control of a virtual hand with high accuracy, speed, and responsiveness. Finally, we present data on the common usability factors of assistive devices including the simplicity, comfortability, portability, and weight of the sleeve. Overall, all subjects reported good to outstanding ratings for each of the usability categories, indicating that the NeuroLife EMG System can provide accurate decoding of upper extremity motor intention while meeting the usability needs of end users.

### Supplementary Information

**Additional file 1.** Supplementary Data.

**Additional file 2. Media 1.** Example of online decoding using NN model in stroke subject 13762. In the top video, an experimenter prompted the user with various movement cues (Hand Close, Hand Open, and Forearm Supination) in a random order. A virtual hand on the computer monitor illustrated the real-time decoded movement intention from each subject’s EMG activity. In the bottom left, a heatmap shows the RMS activation across the sleeve at each timepoint. In the bottom right, a time series plot depicting decoder class probability across time. The presented cue is shown above the time series plot as a rectangular colored bar with the color corresponding to the movement class.

**Additional file 3. Media 2.** Example of online decoding using NN model in stroke subject 30458. Refer to Additional File 2 caption for more details.

## Data Availability

The data that support the findings of this study are available upon reasonable request from the authors.
