# Supplementary material for: Decoding hand and wrist movement intention from chronic stroke survivors with hemiparesis using a user-friendly, wearable EMG-based neural interface
Source: J Neuroeng Rehabil. 2024 Jan 13;21:7. doi: 10.1186/s12984-023-01301-w (PMC10787968; doi:10.1186/s12984-023-01301-w)
Supplement: Supplementary file 1 — Additional file 1. Supplementary Data. [file 12984_2023_1301_MOESM1_ESM.docx]

Supplementary Material

## Supplementary Methods

### Inclusion and Exclusion Criteria

For physically impaired individuals, inclusion criteria address the minimum length of time since the stroke that led to the impairment. Inclusion criteria may also pertain to meeting dimensional requirements related to interacting with the system hardware (e.g., subject’s arm dimensions must be such that they can appropriately don an existing electrode sleeve design).

For populations with potential for cognitive impairment (e.g., stroke survivors), inclusion criteria indicating ability to follow 3-step commands and communicate verbally (e.g., at least able to provide yes/no responses with accuracy) apply.

Specific Inclusion Criteria include:

1. Males and females ≥ 18 years old

2. Chronic stroke survivors who are at least 180 days post-stroke

3. Ability to provide appropriate consent to partake in the study

4. Ability to follow 3-step commands and deemed by an occupational therapist to have the capacity to complete required upper extremity movements

5. Ability to secure transportation to attend scheduled study sessions

6. Stroke-related hand impairment that interferes with ability to complete activities of daily living and is classified as Stage 1-6 on the hand subscale of the Chedoke McMaster Stroke Assessment

Persons with life-supporting or sustaining equipment or critical non-removeable implanted electronic devices are excluded for safety reasons since it is not known if the experimental systems would interfere with this equipment.

Specific exclusion criteria include:

1. Presence of any other clinically significant medical comorbidity for which, in the judgment of the Investigator, participation in the study would pose a safety risk to the subject

2. Currently participating in physical rehabilitation (e.g., occupational or physical therapy) for stroke-related upper limb impairment

3. Co-occurring neurological condition (e.g., Parkinson’s disease, Multiple Sclerosis) or other neuromuscular disorder (e.g., Carpal Tunnel Syndrome, neuropathy) that, in the judgment of the Investigator, may influence study results

4. Individuals who are immunosuppressed, have conditions that typically result in becoming immunocompromised, taking chronic steroids, or currently receiving immunosuppressive therapy

5. Individuals having or requiring any of the following: implanted pacemaker, life supporting/sustaining equipment, or critical non-removable implantable electronic devices such as an insulin pump or neurostimulator. An implanted Medtronic LINQ monitor does not meet this criterion (i.e., patients with a LINQ monitor may participate in this study).

6. Persistent pain ≥ 7/10 in impaired upper extremity, as measured by Numeric Pain Rating Scale (0-10)

7. Individuals whose forearm is determined to be too small or too large to fit the electrode sleeve being investigated.

8. Individuals who are pregnant or plan to get pregnant during the course of the study (self- report).

### Supplementary Figures


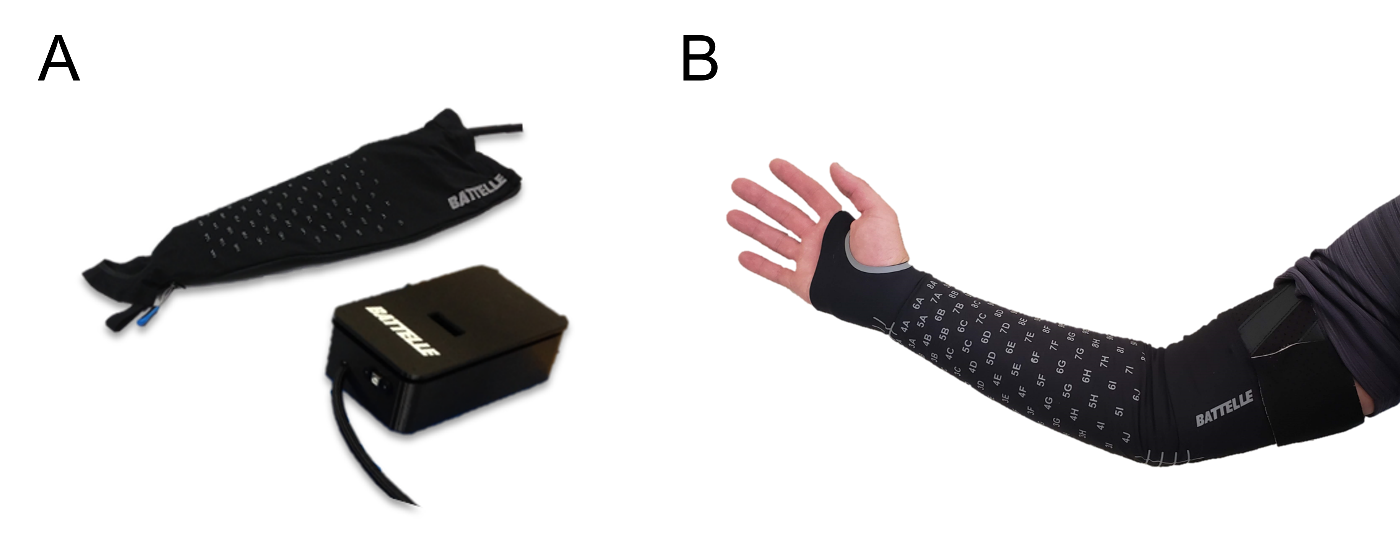


**Supplementary Figure 1. (A)** Configuration of hardware used for EMG data collection showing the sleeve and EMG signal acquisition module (ESAM). **(B)** View of the sleeve donned on a subject.


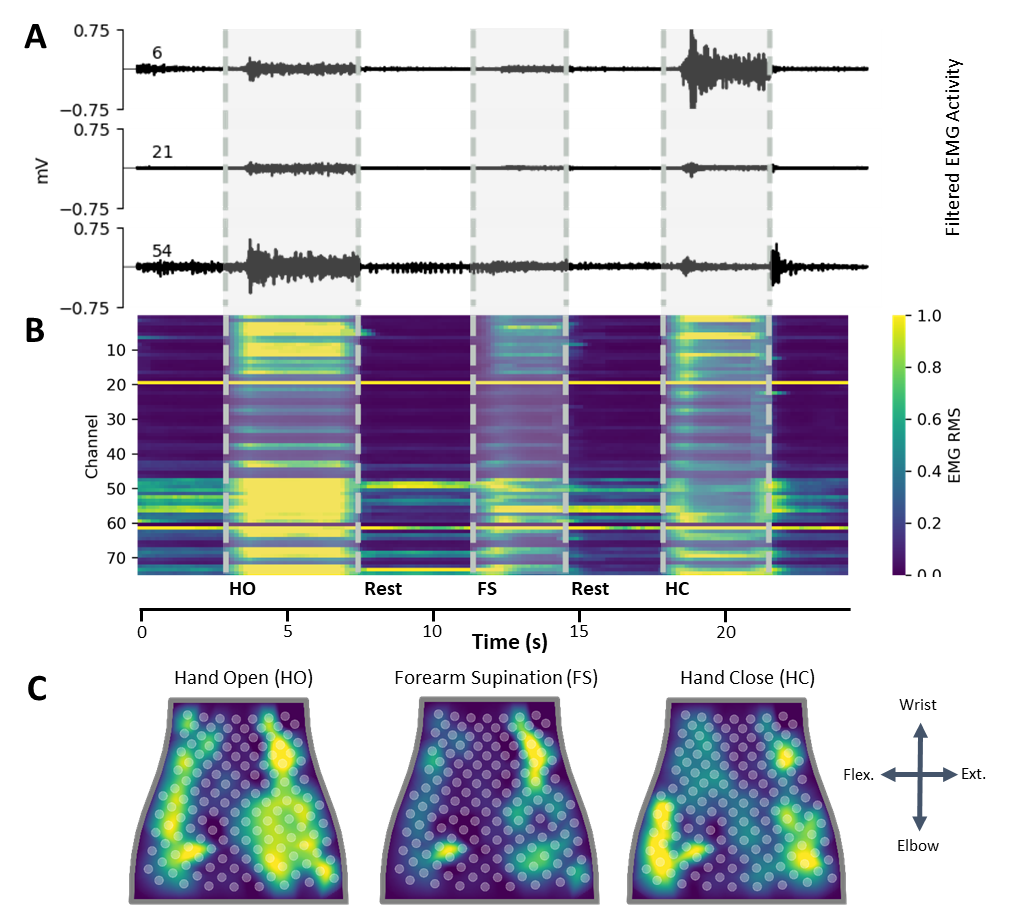


**Supplementary Figure 2. Representative EMG data recorded from able-bodied subject. (A)** Filtered EMG data recorded from 3 separate channels on the NeuroLife Sleeve during 3 movements: Hand Open, forearm supination, and Hand Close. **(B)** Heatmap of normalized RMS activity, with the channel number on the y-axis and time on the x-axis. Note the activity across clusters of electrodes for each of the 3 separate movements. **(C)** Normalized RMS activity mapped to the sleeve orientation, with a legend showing the orientation of the sleeve mapping (flex. = flexors, ext. = extensors). Note the location of EMG activity is spatially located near the related musculature for each of the 3 movements.


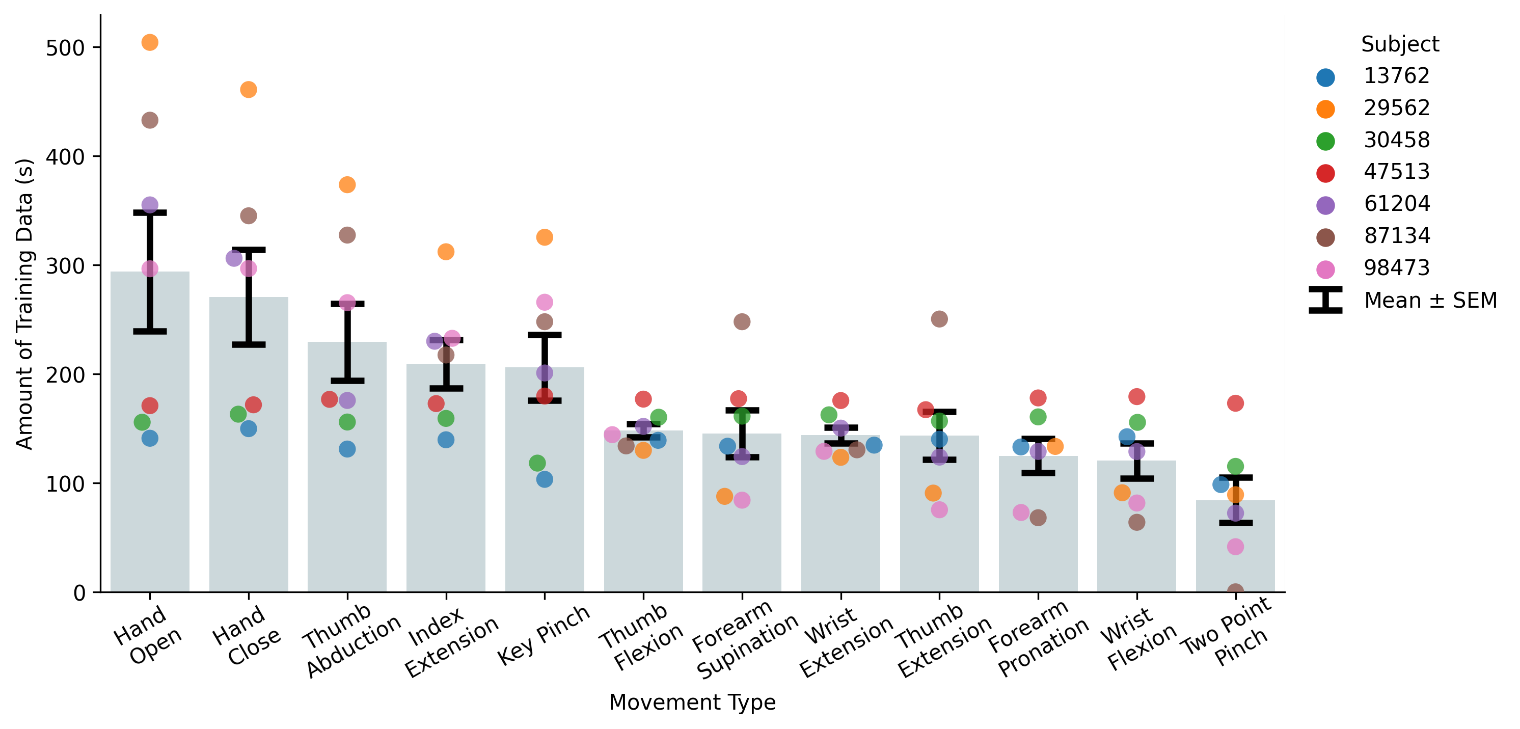


**Supplementary Figure 3.** Amount of EMG data collected in time for each movement type per subject to train the decoders.


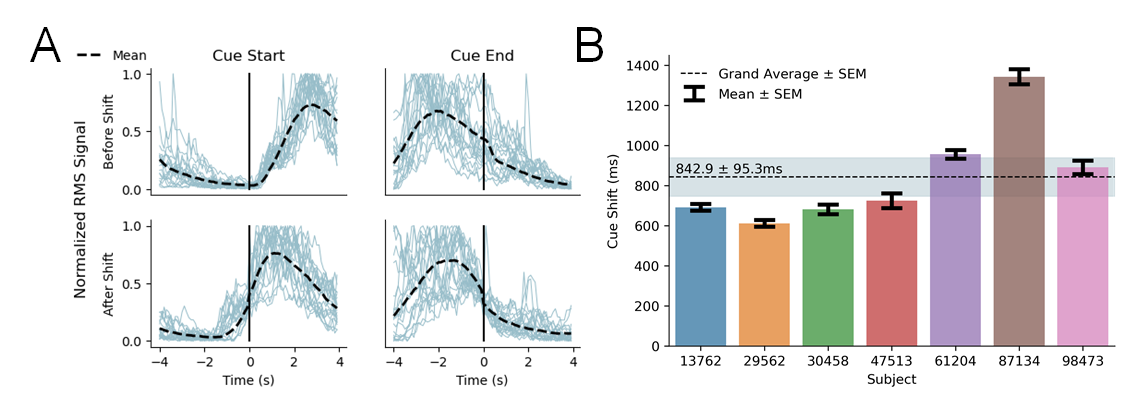


**Supplementary Figure 4. Dynamic cue shifting. (A)** Cue shifting to improve the alignment of RMS of EMG signals during cue start and end times. Without dynamic cue shifting, the detected EMG signal is delayed during cue onset due to reaction time deficits and is sustained through the end of the cue due to residual muscular activity. By shifting dynamically based on underlying EMG activity, we properly align the intended cue with motor intention during training for better decoding performance. **(B)** Average cue shift per subject determined from the dynamic cue shifting technique.


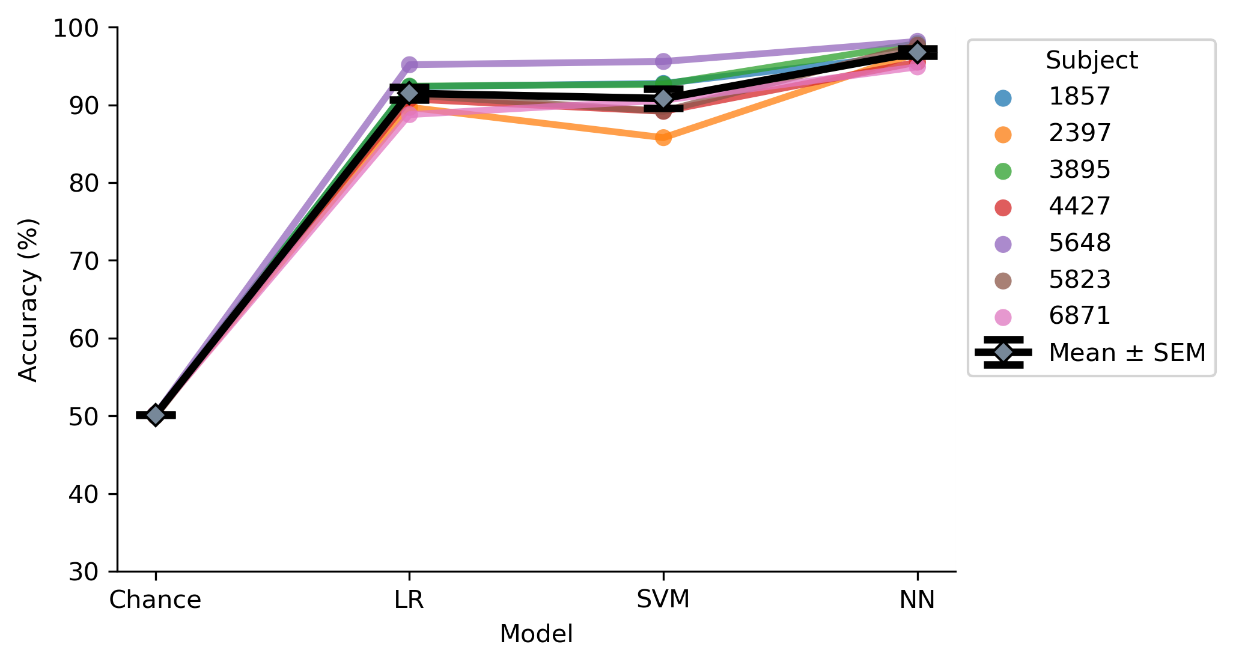


**Supplementary Figure 5.** Model performance for able-bodied subjects comparing 3 models: LR (Logistic Regression), SVM (Support Vector Machine), and NN (Neural Network). The NN outperforms both the LR and SVM models (paired t-test NN vs. LR, p=5.8 x 10^-5^; NN vs. SVM, p= 1.6 x 10^-3^).


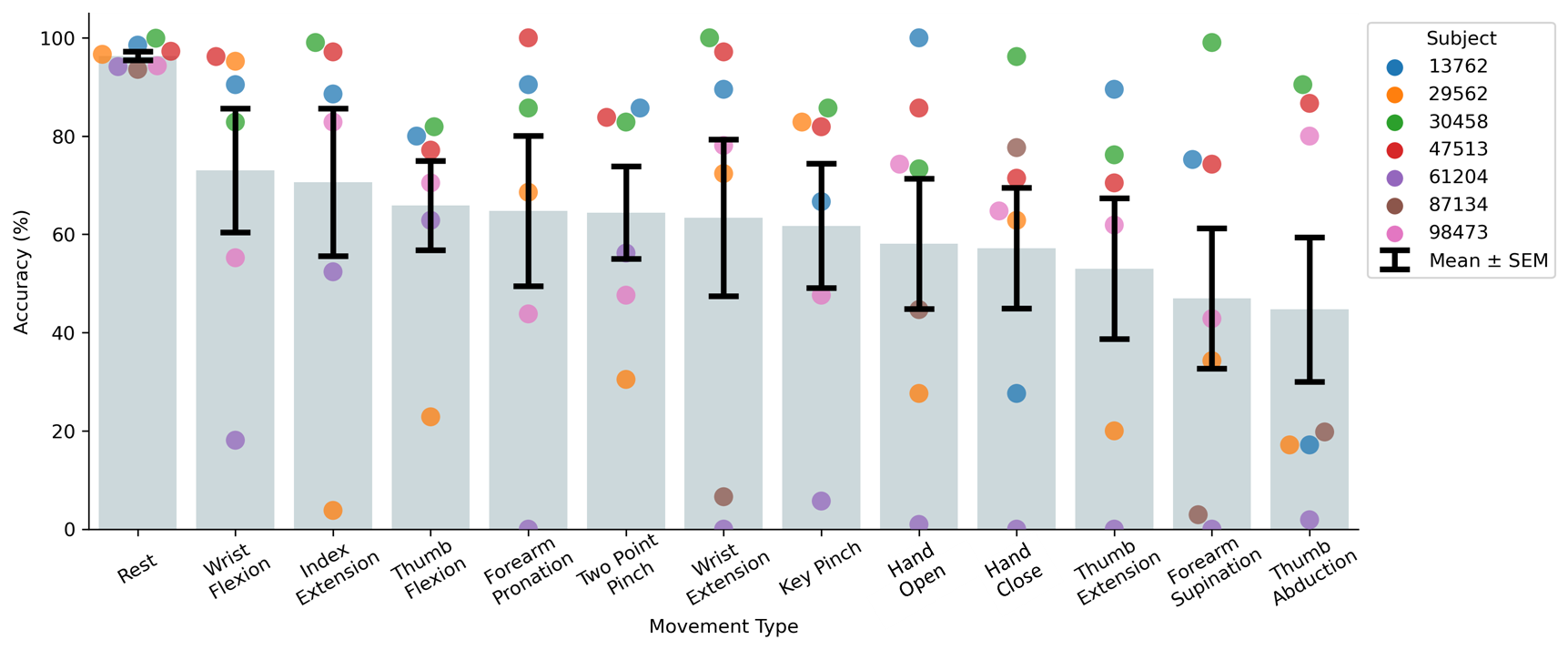


**Supplementary Figure 6.** Decoding performance of the NN model based on movement type per subject.


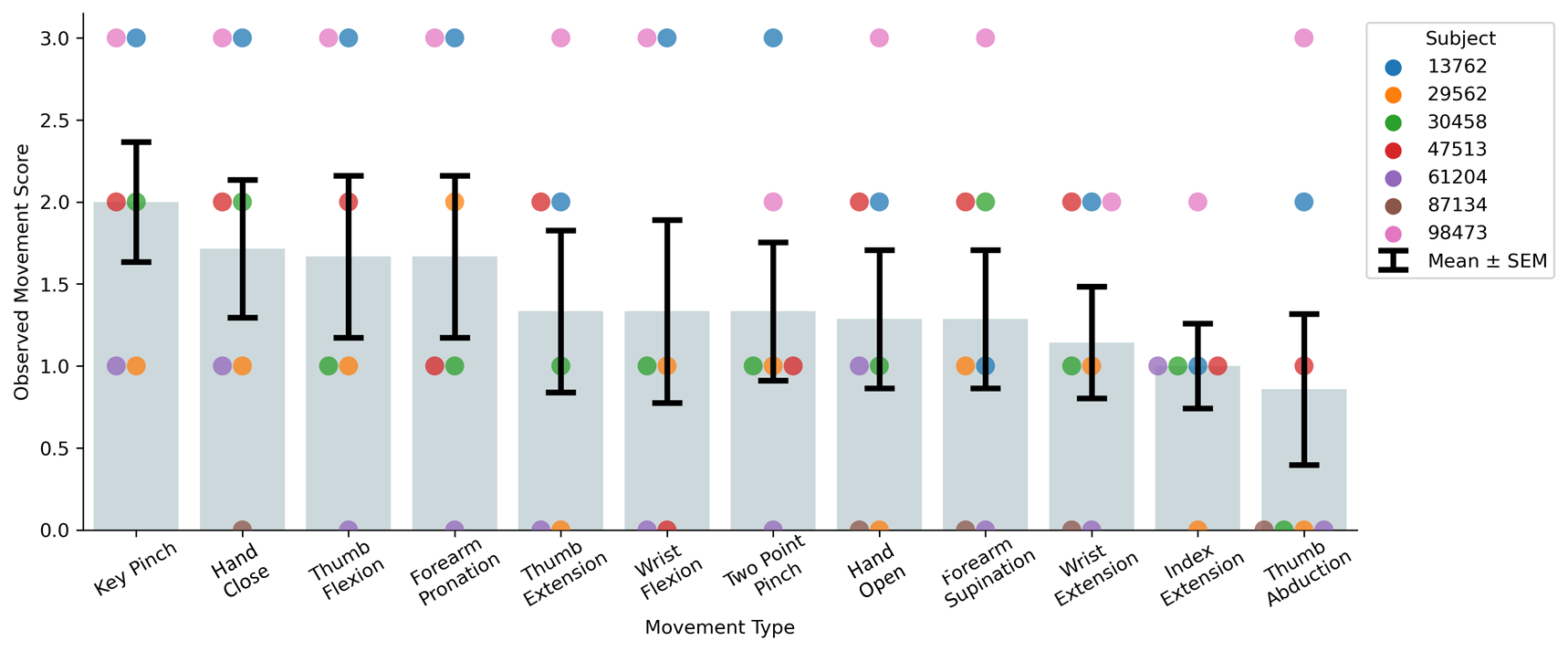


**Supplementary Figure 7.** Observed movement score for each of the functional movements per subject ranked in order of ability from left to right. The Key Pinch and Hand Close were the simplest for subjects to perform whereas more complex movements such as Index Extension and Thumb Abduction were more challenging for stroke participants.


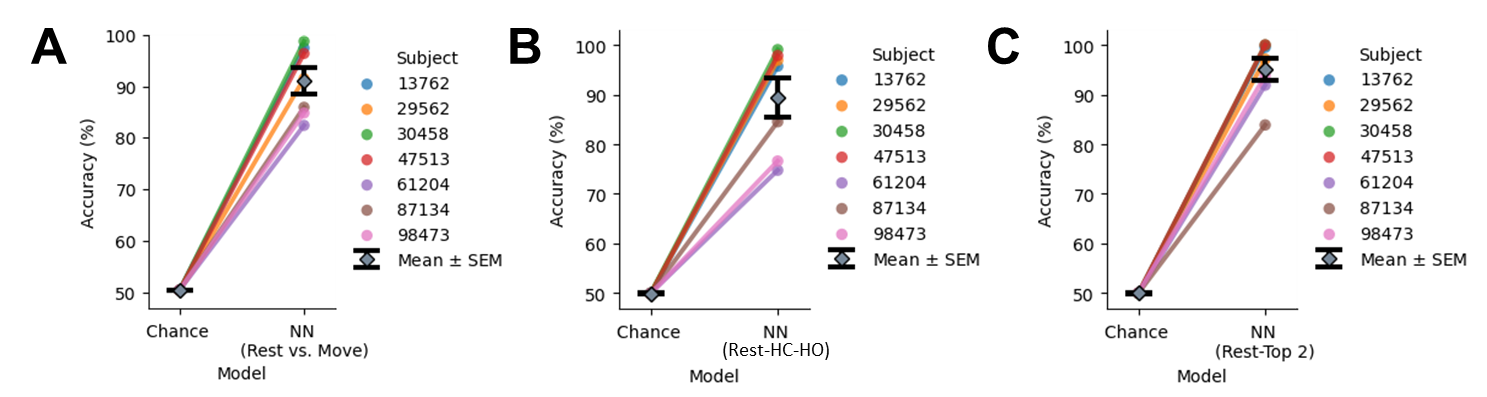


**Supplementary Figure 8. Decoding performance of NN model when down-selecting classes across all subjects. (A)** Decoding performance of NN binary classifier comparing Rest and Move in which Move is made up of combining all 12 movements into a single class. **(B)** Decoding performance of NN model when restricting classes to Rest, Hand Close (HC), and Hand Open (HO). **(C)** Decoding performance of NN model when restricting classes to Rest and the top 2 movements for each subject for a total of three classes.


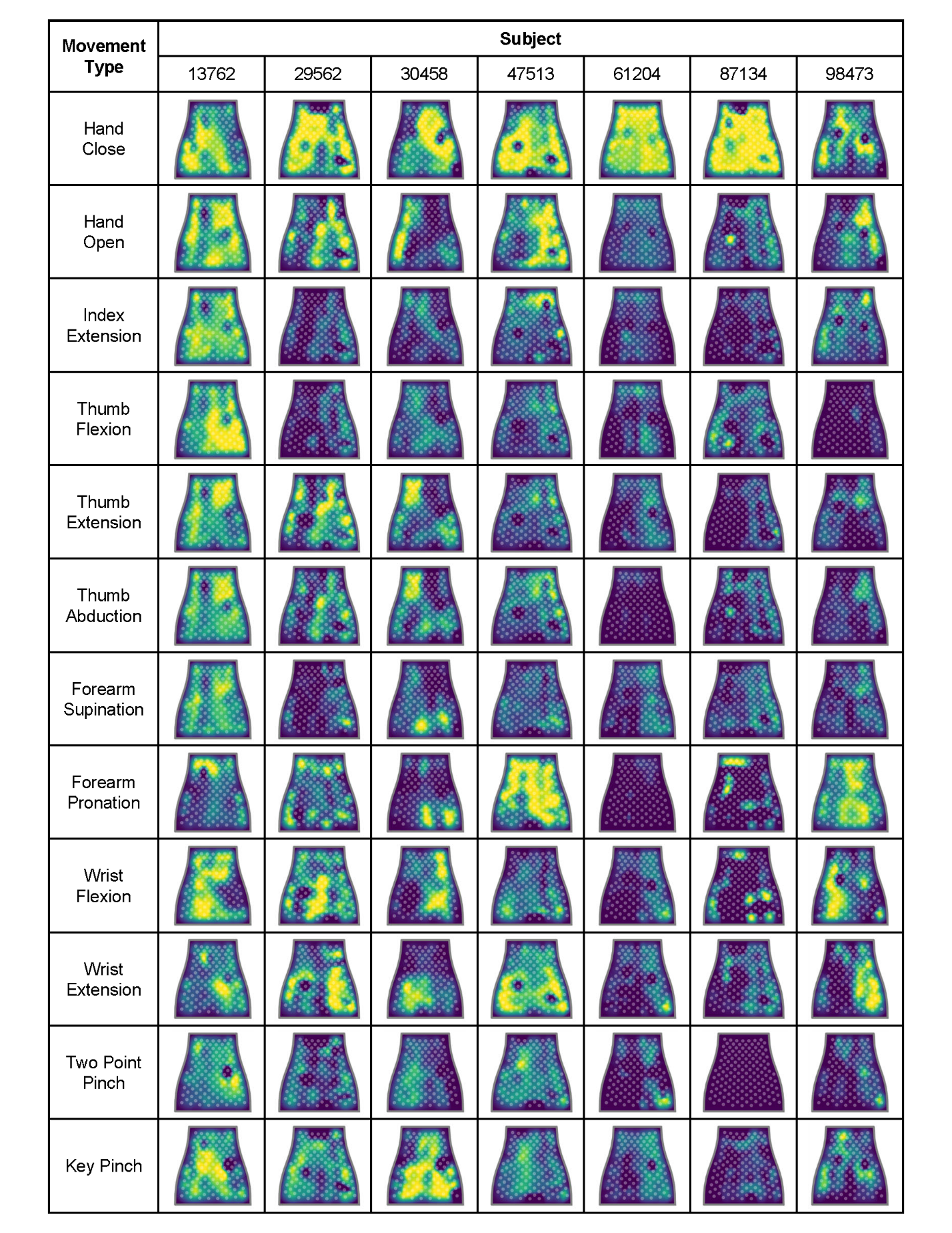


**Supplementary Figure 9.** Normalized RMS activity mapped to the sleeve across all subjects and movements. Refer to Figure 2 for sleeve heatmap orientation.


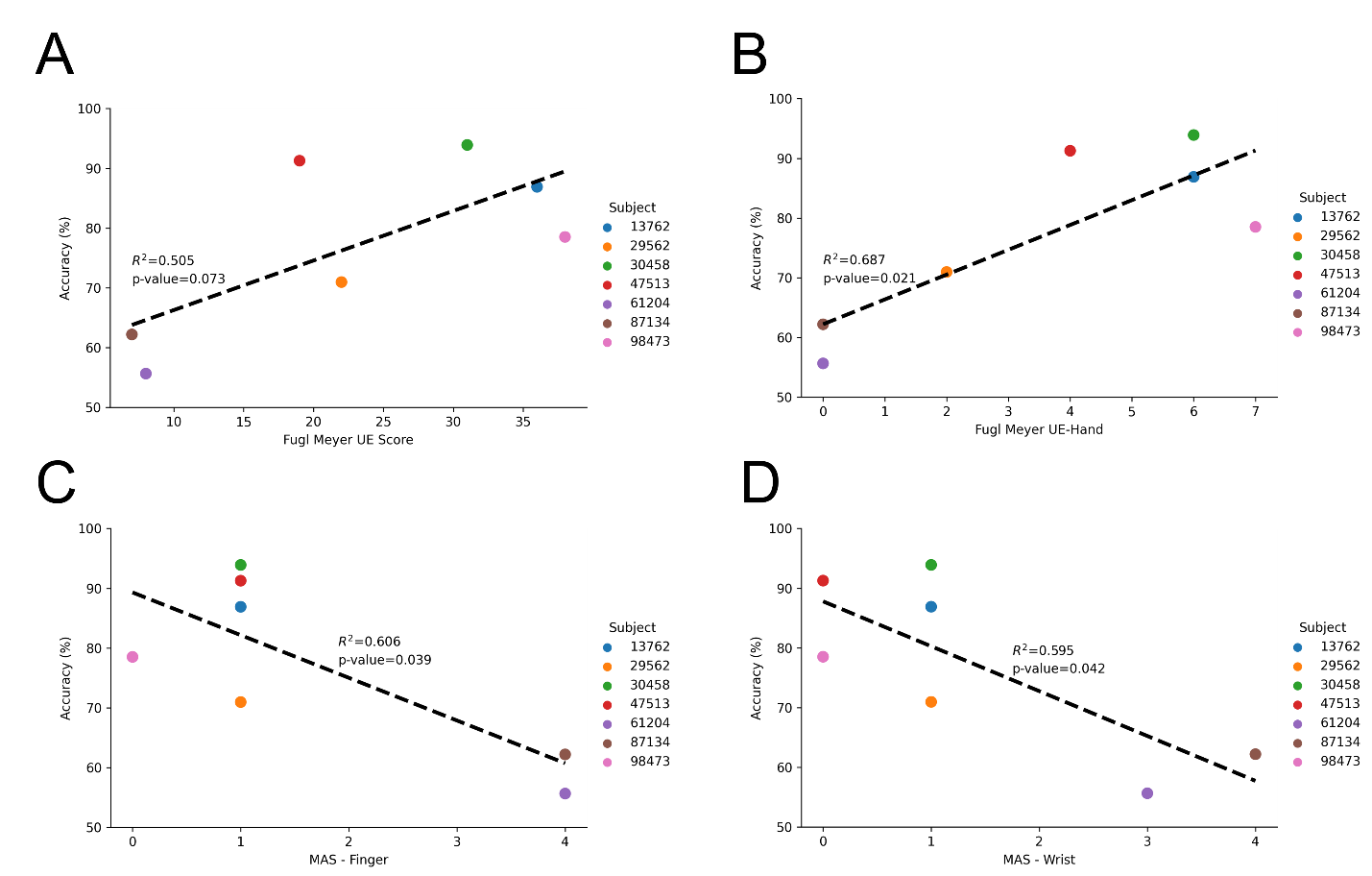


**Supplementary Figure 10.** Scatter plots depicting relationships between overall decoding accuracy (y-axis) and clinical metrics (x-axis). Dashed lines indicate trend lines, with the coefficient of determination (R^2^) and p-value reported on each plot determined using the Wald Test.

|  | **Small** | **Medium** | **Large** |
| --- | --- | --- | --- |
| **Number of Electrodes** | 128 | 142 | 150 |
| **Number of Electrode Pairs** | 64 | 71 | 75 |
| **Weight (grams)** | 180 | 195 | 220 |

**Supplementary Table 1. Characteristics of the 3 different sized NeuroLife Sleeve.**

| **Participant** | **Age Range, years** | **Sex** |
| --- | --- | --- |
| 1857 | 26-30 | Male |
| 2397 | 31-35 | Female |
| 3895 | 26-30 | Female |
| 4427 | 26-30 | Male |
| 5648 | 21-25 | Female |
| 5823 | 21-25 | Female |
| 6871 | 26-30 | Male |

**Supplementary Table 2. Demographics of able-bodied subjects.**

| **Group 1 Movement Score** | **Group 2 Movement Score** | **Mean Accuracy Difference (%)** | **P-value** | **Reject** |
| --- | --- | --- | --- | --- |
| 0 | 1 | 38.2 | 0.001 | True |
| 0 | 2 | 49.7322 | 0.001 | True |
| 0 | 3 | 46.5521 | 0.001 | True |
| 1 | 2 | 11.5323 | 0.5262 | False |
| 1 | 3 | 8.3521 | 0.703 | False |
| 2 | 3 | -3.1802 | 0.9 | False |

**Supplementary Table 3. Tukey HSD multiple comparison results of decoded accuracy vs. movement ability.** Subjects not able to perform the movement at all (Movement score=0) had a statistically significant difference in decoding performance compared to movements with scores ≥ 1.

| **Subject** | **Top Movements: Successes/Attempts** | | |
| --- | --- | --- | --- |
| 29562 | Rest: 10/10 | Key Pinch: 5/5 | Wrist Flexion: 5/5 |
| 61204 | Rest: 10/10 | Two Point Pinch: 5/5 | Index Extension: 4/5 |
| 87134 | Rest: 24/26 | Hand Close: 11/13 | Hand Open: 7/13 |

**Supplementary Table 4. Success rate of top performing movements in subjects with severe hand impairment (UEFM-HS < 3).**

| Scale 1-5 (1 bad, 5 great) | Domain | 29562 | 98473 | 30458 | 47513 | 61204 | 87134 | 13762 |
| --- | --- | --- | --- | --- | --- | --- | --- | --- |
| How simple was the sleeve to put on with our help? (1 = not simple, 5 = extremely simple) | Simple to apply | 1 | 5 | 4 | 3 | 3 | 5 | 5 |
| How simple was the sleeve to take off with our help? (1 = not simple, 5 = extremely simple) | Simple to apply | 1 | 5 | 5 | 5 | 4 | 4 | 5 |
| How confident are you that a caregiver could help you put the sleeve on properly? (1 = not confident, 5 = extremely confident) | Simple to apply | 4 | 5 | 3 | 4 | 5 | 3 | 5 |
| How confident are you that a caregiver could help take off the sleeve properly? (1 = not confident, 5 = extremely confident) | Simple to apply | 4 | 5 | 4 | 5 | 5 | 3 | 5 |
| How confident are you that you could put the sleeve on by yourself properly? (1 = not confident, 5 = extremely confident) | Simple to apply | 1 | 2 | 2 | 2 | 3 | 1 | 3 |
| How confident are you that you could take the sleeve off by yourself properly? (1 = not confident, 5 = extremely confident) | Simple to apply | 3 | 3 | 3 | 5 | 4 | 1 | 3 |
| How comfortable was the sleeve during the two-hour session (1 = extremely uncomfortable, 5 = extremely comfortable)? | Comfortability | 4 | 5 | 5 | 5 | 5 | 4 | 4 |
| How much did the sleeve restrict your normal arm and hand movement (1 = extremely restrictive, and 5 = not restrictive)? | Freedom of movement | 4 | 4 | 5 | 3 | 5 | 3 | 4.5 |
| How confident are you that you could wear the sleeve doing light activities around your home if the sleeve is wireless (1 = not confident, 5 = extremely confident)? | Functionality / lightweight / portability | 5 | 4 | 4 | 4 | 2 | 3 | 5 |
| How likely would you be to wear the sleeve for a long duration during the day (more than 2 hours) (1 = extremely unlikely, 5 = extremely likely)? | Functionality / lightweight / portability | 4 | 4.5 | 5 | 5 | 5 | 3 | 3.5 |
| Would you want to use this system with a therapist (1 = you wouldn’t want to use it, 5 = you would really want to use it)? | General favorability | 5 | 5 | 5 | 4 | 5 | 5 | 5 |
| Would you want to use this system in your home (1 = you wouldn’t want to use it, 5 = you would really want to use it)? | General favorability | 3 | 5 | 5 | 5 | 5 | 5 | 5 |
| How do you like the overall design of the device (look, feel, comfort, etc.)? (1=I hate it, 5=I love it) | Aesthetics / Design | 5 | 5 | 4 | 4 | 5 | 4 | 3.5 |

**Supplementary Table 5.** Complete usability questionnaire responses from all subjects with stroke in the study. Each question was categorized to a specific domain, shown in the domain column and this data was used in Figure 7.

| **Normalization Method** | **Accuracy (Mean ± SEM)** |
| --- | --- |
| Concatenate session data and then normalize (mean=0, variance=1). Testing data normalized using mean and variance from concatenated data. This method was used in Figure 5A-Dynamic. | 74.7 ± 5.0% |
| Normalize data from each session (mean=0, variance=1) then concatenate sessions. Testing data normalized using mean and variance from within session training data. | 74.7 ± 5.0% |
| Train on within session data only. Normalize (mean=0, variance=1) training data within session. Testing data normalized using mean and variance from within session training data. | 73.9 ± 5.2% |

**Supplementary Table 6.** Comparison of training dataset normalization methods on decoder accuracy.

**Supplementary Media 1.** Example of online decoding using NN model in stroke subject 13762. In the top video, an experimenter prompted the user with various movement cues (Hand Close, Hand Open, and Forearm Supination) in a random order. A virtual hand on the computer monitor illustrated the real-time decoded movement intention from each subject’s EMG activity. In the bottom left, a heatmap shows the RMS activation across the sleeve at each timepoint. In the bottom right, a time series plot depicting decoder class probability across time. The presented cue is shown above the time series plot as a rectangular colored bar with the color corresponding to the movement class.

**Supplementary Media 2.** Example of online decoding using NN model in stroke subject 30458. Refer to Supplementary Media 1 caption for details.
